# Fine structural tuning of the assembly of elastin–collagen peptide conjugates with drug loading and manipulation of molecular interactions

**DOI:** 10.1039/d5bm01470k

**Published:** 2026-05-22

**Authors:** Haofu Huang, Jingya Qin, Sirui Shen, Jeongmin Hwang, Darrin J. Pochan, Kristi L. Kiick

**Affiliations:** a Department of Materials Science and Engineering, University of Delaware Newark DE USA pochan@udel.edu; b Department of Biomedical Engineering, University of Delaware Newark DE USA

## Abstract

Elastin–collagen nanoparticles (ECnPs) have been shown in our previous studies to self-assemble into different morphologies, including nanoplates and nanovesicles, by manipulating the sequence length of the elastin-like peptide (ELPs) and collagen-like peptide (CLPs) of a given conjugate. In this work, we demonstrate that the morphologies of ECnPs can also be modulated, for a given ECnP sequence, with variations in solution pH and/or the amount of encapsulated drug. Specifically, the peptide (VPGYG)_6_-(GPO)_8_ preferentially formed nanovesicles under basic conditions but assembled into nanoplates under acidic conditions. Another sequence, (VPGWG)_2_(VPGFG)_2_-(GPO)_8_, produced nanovesicles when loaded with a high concentration of dexamethasone-carboxyfluorescein (Dex-CF), but transitioned to nanoplates at lower drug loading. Furthermore, in addition to the different morphologies observed for a given set of initial solution conditions, our studies also illustrate the possibility of triggering vesicle-to-plate transformations for a given ECnP with release of Dex-CF over time. These results highlight multiple avenues for controlling ECnP morphology, expanding their applicability as a flexible and efficient drug delivery platform.

## Abstract 2

Two elastin–collagen peptide conjugates whose self-assembled shapes are tuned between spherical and platelet by the aqueous solution conditions. Y_6_-G_8_ nanoparticle was tuned by the pH, and the W_2_F_2_-G_8_ nanoparticle was tuned by the Dex-CF amount.

## Graphical abstract

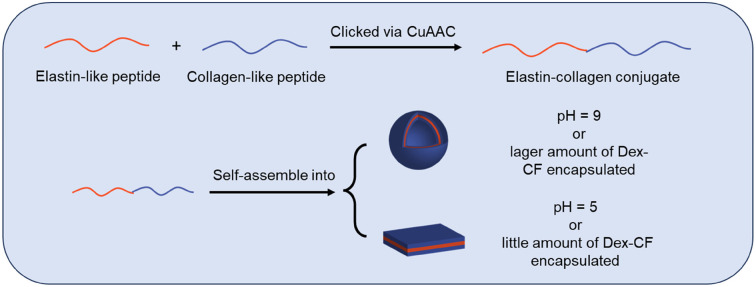

## Introduction

For decades, the self-assembly of amphiphilic molecules has been a popular approach in bioengineering, with its applications extending to the design of advanced drug delivery systems and substrates for tissue engineering.^1–3^ Specifically, peptide-based conjugates have been identified as beneficial due to their modifiable secondary structures, self-association tendencies, biodegradability, and biocompatibility.^4–6^ By manipulating the chemical structure, molecular geometry, solvent conditions, dimensions, and interfacial curvature of peptide-inclusive amphiphiles, the morphologies of self-assembled structures can be controlled. Furthermore, establishing systematic design paradigms that facilitate structural modifications *via* minor sequence adjustments significantly enhances the possibility of expanding their practical applications. This has been further optimized by the integration of stimuli-responsive domains that demonstrate significant property transformations in response to subtle environmental adjustments, such as changes in temperature, pH, or solvent conditions.^6–8^ Notably, thermo-responsiveness is a frequently employed property due to its straightforward application and capacity to modulate peptide conformations such as alpha-helices, beta-sheet, and coiled coils.^5,9^ Thermo-responsive peptide-amphiphilic copolymers are thus versatile tools for designing drug delivery carriers with tunable properties.

Nanoscale drug delivery systems (NDDs) have been widely used to achieve effective treatment and diagnosis of diseases.^10–12^ As mentioned above, peptide-based NDDs not only share the advantages of traditional nanomedicine approaches but also exhibit extensive synthetic flexibility to induce biological function and responsiveness.^13,14^ NDDs have been a key tool for improving controlled drug delivery, mainly by overcoming pharmacokinetic limitations (*e.g.*, rapid clearance) observed for free drugs. The improved efficacy has been achieved through the localization of drugs to the specific sites with passive/active targeting strategies and the release of the drugs in a controlled manner *via* endogenous and exogenous stimuli.^15–17^ Owing to their nanoscale dimensions (ranging from 10 to 1000 nm), vehicles can extravasate from circulation and penetrate tissue to accumulate drugs passively at target sites such as tumors,^18^ inflammatory sites,^19^ and atherosclerotic plaques.^20^ Moreover, many carriers can be actively targeted to diseased sites *via* the chemical conjugation to their surfaces of targeting moieties such as antibodies and peptides; modified nanoparticles then can interact selectively with receptors overexpressed or expressed only at the targeted sites.^21^ As a widely and long-studied example, RGD peptides, derived from multiple extracellular matrix proteins, can be attached to the surface of polymeric micelles and used to target the upregulated αvβ3 integrin at tumor sites.^22^ In addition to the targeting effect of the drug delivery system, peptide conjugation also prolongs blood circulation and improves bioavailability. Taken together, these features result in the reduction of side effects and increased efficacy of drugs.^18,21–24^

In addition to their dimension, the shape of a drug carrier is also a critical factor that influences *in vivo* pharmacokinetics and pharmacodynamics, as well as cellular uptake.^25–27^ Spherical nanoparticles have been studied extensively as drug delivery systems based on the advantages of their properties including chemical versatility, high surface-to-volume ratio, and unique optical properties.^26^ In addition to traditional spherical nanoparticles, non-spherical nanostructures can also be advantageous in drug delivery, particularly for intracellular targets, as they exhibit a greater propensity for uptake by target cells.^28^ This has been attributed to the fact that differences in nanostructure shape cause changes in surface curvature, attachment to cells, and thus overcome biological barriers which directly influences cellular internalization.^23,29^ For example, PAMAM-*b*-OEG co-dendrimer-based nanosheets,^24^ rod-shaped mesoporous silica nanoparticles,^25,30^ short-rod nanoparticles,^31,32^ and worm-like filo-micelles^27,33,34^ all show improved uptake and drug delivery efficiency over their spherical counterparts, despite having similar dimensions and drug release profiles. These differences also affect pharmacokinetics and pharmacodynamics; indeed, rod-like nanoparticles and sheet nanostructures have exhibited higher cell uptake, delayed clearance, and longer circulation times.^30–32^ For PLGA-based nanoparticles, for example, ellipsoidal particles were phagocytosed at a lower rate and a lower inhibition of particle uptake compared with spherical nanostructures,^35,36^ confirming the impact of shape/morphology of carriers on the fate of the drugs.

However, opportunities remain to understand in more detail the impact of drug vehicle morphology on delivery, and peptide-based drug delivery systems offer significant potential in this regard, as their assembly can be finely tuned for specific applications, including by altering cargo content. Peptides derived from naturally occurring self-assembly motifs (*e.g.*, α-helix, β-sheets, and coiled-coils) in proteins enable the self-assembly of nanostructures including nanotubes, nanovesicles, nanodonuts, nanofibers, nanoparticles, and spherical or cylindrical micelles.^37–41^ Because the self-assembly of peptides is driven by an interplay of various non-covalent forces (*e.g.*, electrostatic interactions, hydrophobic interactions, hydrogen bonding, and π–π stacking^42,43^), resulting morphologies of self-assembled peptide nanostructures can be easily manipulated by tuning features of the peptides, or by changing external triggers (*e.g.*, temperature, solvent, or pH) or potentially *via* the interactions with additional chemical compounds (*e.g.*, drugs).^44^ Therefore, the morphological adjustment of self-assembled peptides with drug loading presents a significant opportunity to explore how both the morphology of a drug delivery system as well as drug types influence drug delivery efficiency.^43–50^

Our group has developed thermoresponsive nanostructures of various morphologies using elastin-like peptide (**ELP**) and collagen-like peptide (**CLP**) conjugates (**ECC**s).^43,49,51–53^ ELPs comprising the pentapeptide repeat (VPGXG, X = any amino acid, except P) exhibit lower critical solution temperature (LCST)-like behavior (also termed an inverse transition temperature, *T*_t_), which enhances ELP coacervation and has been used as a building block for the assembly of nanostructures. However, the short ELPs possess a high transition temperature inappropriate for use in biomedical applications. To overcome this limitation, our group utilized the triple helical folding of the ELP-equipped CLP to reduce the inverse transition temperature of the ELP within the physiological range. The CLPs most commonly employed have been (GPO)_*n*_ (O is hydroxyproline). The resulting ELP–CLP nanoparticles (ECnPs), including ELP–CLP nano-vesicles (ECnVs), have demonstrated targeting to collagen *via* CLP strand invasion, the release of cargo in response to temperature, and cytocompatibility,^51^ consistent with other studies in which the systemic delivery of caged CLP resulted in accumulation prominently in bone, articular cartilage, and tumors after photo-triggered removal of the cage and subsequent CLP triple helix formation.^54^ This strategy has also been applied for targeting native collagen in mouse skin *in vitro* and *in vivo*.^55^ In addition to the ability of CLP for targeting native collagen and the controlled delivery of cargo,^56^ ECnPs enable *dual* thermo-responsive release of cargo. The release of cargo can be controlled by the manipulation of both *T*_t_ and *T*_m_ of the ECC, which can be easily achieved by the modification of ELP and CLP domains.

While our earlier research shed light on the capability of modifying assembled structures of ECnPs by manipulating the domain lengths of different sequences, the possibility of creating a range of morphologies from a single sequence of ECnP has not been explored,^57,58^ despite the known and crucial role, as mentioned above, that diverse morphologies can play in optimizing cellular uptake and drug delivery regulation. Managing carrier structures could offer substantial promise for tailoring carriers for specific drugs and their respective applications. Furthermore, it's plausible to engineer ECnPs with additional capabilities by integrating bioactive peptides, thereby augmenting cell penetration, targeting specificity, release mechanisms, and facilitating endosomal escape.^58,59^

In this report, we present the impact of pH and drug loading of select ECnP constructs. Because of the ability to ionize tyrosine at various pH values, we elected to study the impact of pH on the morphologies adopted by tyrosine-containing ECnPs under different pH conditions. An ECC with the sequence (VPGYG)_6_-(GPO)_8_ (**Y**_**6**_**-G**_**8**_ in short), which adopts a vesicle-like structure in slightly basic solutions, was used to study the influence of solution condition (pH, surface charge) on the assembled structures of the ECnPs. In addition, because our previous studies illustrated the sensitivity of **W**_**2**_**F**_***m***_**-G**_***n***_, which represents (VPGWG)_2_(VPGFG)_*m*_–(GPO)_*n*_ constructs to adopt spherical or plate-like morphologies, we sought to determine in the present work if drug loading could also facilitate similar changes in morphology. An ECC with the sequence W_2_F_2_-G_8_, which adopts a plate-like nanostructure at slightly elevated temperatures, was loaded with the hydrophobic cargo dexamethasone-carboxyfluorescein (Dex-CF) to study the influence of the model drug on the assembled structures of the ECnPs. Electrospray ionization mass spectrometry (ESI-MS) and high-performance liquid chromatography (HPLC) were used to confirm the purity of the ECCs, and the conformational and self-assembly behavior was characterized *via* circular dichroism (CD) spectroscopy, dynamic light scattering (DLS), and transmission electron microscopy (TEM). Under different pH conditions, Y_6_-G_8_ self-assembles into different morphologies. With sufficient loading of the model drug Dex-CF, W_2_F_2_-G_8_ self-assembles into vesicles instead of plate-like structures. These studies demonstrated the ability of ECnPs to show reversible morphological transitions between vesicles and plate-like structures under different conditions, and initially suggest the potential to control morphological transitions during the drug release progress.

## Methods and materials

### Materials

Fmoc-protected amino acids (including Fmoc-propargyl glycine), *N*,*N*,*N*′,*N*′-tetramethyl-*O*-(^1^H-benzotriazol-1-yl) uronium hexafluorophosphate (HBTU), and piperidine, for solid-phase peptide synthesis, were purchased from AAPPTEC Inc (Louisville, KY). Rink amide polystyrene resin for solid-phase peptide synthesis was purchased from CEM corporation (Matthews, NC). HPLC-grade acetonitrile and dimethylformamide (DMF) was purchased from Fisher Scientific (Fairlawn, NJ). 4-Azidobutanoic acid, trifluoroacetic acid (TFA), triisopropylsilane (TIS), triethylamine (TEA), anhydrous dimethylformamide (DMF), anhydrous dimethyl sulfoxide (DMSO), diisopropylethylamine (DIEA), ethyl cyanohydroxyiminoacetate (Oxyma), copper(ii) sulfate (Cu(ii)sulfate), (+)-sodium l-ascorbate and diisopropylcarbodiimide (DIC) were purchased from Sigma-Aldrich (St. Louis, MO). Tris-hydroxypropyltriazolylmethylamine (THPTA) was purchased from Click Chemistry Tools LLC (Scottsdale, AZ).

### Peptide synthesis

As described in our previous reports,^43^ collagen-like peptides with the sequence (G̲PO)_8_GG, (G̲PO)_8_GG-COOH (denoted as G_8_ and G_8_-COOH) and elastin-like peptides with sequences (VPGY̲G)_6_G′ (Y_6_), (VPGW̲G)_2_(VPGF̲G)_2_G′ (W_2_F_2_) (G′: propargyl glycine) were synthesized *via* traditional solid-phase peptide synthesis methods (SPPS) using a Liberty Blue™ automated microwave peptide synthesizer (CEM Corporation, Charlotte, NC). Gly-preloaded Wang resin with a loading of 0.49 mmol g^−1^ was used for the synthesis of G_8_-COOH, and the rest of the peptides were synthesized using Rink amide ProTide resin with a loading capacity of 0.19 mmol g^−1^. Oxyma was used to activate the amino acids for coupling in the presence of 1 M DIC in DMF. Deprotection of the Fmoc group was conducted using 20% piperidine in DMF. Double coupling of each amino acid at 90 °C for 10 min with a 4 : 1 amino acid/resin ratio was used for the conjugation reactions. For the CLP domain, 4-azidobutanoic acid then was manually attached to the N-terminus of the CLP while on resin. The alkyne group from propargyl glycine was introduced to the C-terminus of the ELP sequence during the solid-phase synthesis. Cleavage of these peptides from the resin was conducted in 92 : 4.5 : 2.5 (v : v : v) trifluoroacetic acid (TFA)/triisopropylsilane (TIS)/water for 2 hours. The TFA was evaporated under the flow of nitrogen for 30 min and the cleaved peptide was precipitated in cold ether. The peptide was then redissolved in water and purified *via* reverse-phase HPLC (Waters Inc., Milford, MA) on a Waters™ X bridge BEH130 prep C-18 column heated at 60 °C with a UV detector at 214 nm and was then collected and lyophilized. The purity of the peptide was confirmed *via* ultra-performance liquid chromatography in line with electrospray ionization mass spectrometry on a Xevo G2-S QTof (denoted UPLC-MS) (Waters Corporation, Milford, MA). All samples were dissolved in ACN/water solution at room temperature with a concentration of 100 μM. Observed results for the synthesized peptides were as follows: Y_6_ (2952.2 Da (Theo), 2951.9 Da(Exp)); G_8_ (2380.4 Da (Theo), 2380.2 Da (Exp)); G_8_-COOH (2381.4 Da (Theo), 2381.0 Da (Exp)); W_2_F_2_ (2021.2 Da (Theo), 2021.9 Da (Exp)) (Fig. S1–S4).

### ECC synthesis

The synthesis of the ECCs was performed *via* copper(i)-mediated azide–alkyne cycloaddition reaction.^53,60^ THPTA (30.4 mg) was dissolved in 200 μL H_2_O. Cu(ii)sulfate (3.2 mg) was dissolved in 100 μL H_2_O, and sodium l-ascorbate (79.2 mg) was dissolved in 200 μL H_2_O. 6 μ moles of CLP (G_8_ or G_8_-COOH) and 3 μ moles of ELP (Y_6_ or Y_6_) were weighed and transferred to a 10 mL reaction vial. 100 μL THPTA solution, 30 μL Cu(ii) solution, and 200 μL sodium solution prepared above were added to the vial. Then, 370 μL water and 300 μL DMSO were added, at a ratio of water/DMSO of 7 : 3 and a total volume of 1 mL. The reaction was carried out for 1 hour with constant stirring at 90 °C, which is above the unfolding temperature of the CLP domain. The peptide was then purified *via* reverse-phase HPLC (Waters Inc., Milford, MA) on a Waters™ X bridge BEH130 prep C-18 column heated at 60 °C with a UV detector at 214 nm, and it was then collected and lyophilized. As above, the purity of the peptide was confirmed *via* ultra-performance liquid chromatography in line with electrospray ionization mass spectrometry on a Xevo G2-S QTof (denoted UPLC-MS) (Waters Corporation, Milford, MA). All samples were dissolved in ACN/water solution at room temperature with a concentration of 100 μM. The observed results for the ECCs were as follows: Y_6_-G_8_-COOH [5334.74 Da (Theo), 5334.71 Da (Exp)], Y_6_-G_8_ [5333.74 Da (Theo), 5333.59 Da (Exp)], W_2_F_2_-G_8_ [4401.56 Da (Theo), 4401.12 Da (Exp)] (Fig. S5–S7).

### Circular dichroism spectroscopy (CD)

Circular dichroism spectroscopy (on a Jasco 1000 circular dichroism spectropolarimeter, Jasco Inc., Easton, MD) was conducted for the characterization of the secondary structure of the CLP domain. CLP and ECC were dissolved at a concentration of 100 μM in 1× PBS (10 mM, pH 7.4, 137 mM NaCl, and 2.7 mM KCl) and incubated at 4 °C overnight before measurement. The CD spectra were recorded using quartz cells with a 0.1 cm optical path length. Full wavelength scans were collected to study the conformation of the peptide domain at 4 °C. The scanning rate was 50 nm min^−1^, with a response time of 4 s. The wavelength scans were obtained from 200 to 250 nm and were recorded every 1 nm. To measure the melting temperature of the CLP domain, variable temperature experiments were conducted at the maximum wavelength in each ELP−CLP conjugate (*e.g.*, 224 nm) with a 6 °C h^−1^ heating rate from 4 °C to 80 °C. Boltzmann fitting of the melting curve was conducted and the corresponding temperature with the highest first derivative was defined as the melting temperature (*T*_m_).

### Dynamic light scattering (DLS)

Dynamic light scattering (DLS) was conducted with a ZetaSizer Nano Series (Nano ZS, Malvern Instruments, UK) to analyze particle diameters in solutions of ECnPs and Dex-CF-loaded ECnPs. Measurements were collected at a scattering angle of 173°, and data were fit using the cumulant method. The ECnP was dissolved in an aqueous solution at 0.5 mg mL^−1^ at room temperature and was incubated at 80 °C in an oven for 2 hours to unfold the CLP, followed by cooling the sample from 80 °C to ambient temperature on the laboratory bench and incubation at ambient temperature overnight. The preparation procedures for the Dex-CF-loaded samples were conducted in the dark (and/or with foil-covered glassware). As described above, 5 mg Dex-CF was dissolved in 200 μL ethanol and the ethanol solution was split into three separate Dex-CF solutions with final Dex-CF concentrations of 25 mg ml^−1^, 4.2 mg mL^−1^, and 2.5 mg mL^−1^. These Dex-CF solutions (10 μL) were added to 490 μL deionized water (DI water) of an ECC solution (0.25 mg in 490 μL DI water) to yield three different mass ratios of Dex-CF : ECC of 1 : 1, 1 : 6 and 1 : 10 respectively. In order to load the Dex-CF, the ECC solutions were incubated at 80 °C for 2 hours to unfold the CLP and solubilize the ECC. 10 μL Dex-CF solution was added to the ECC solution, and the solution of ECC with Dex-CF was cooled from 80 °C to 25 °C and incubated at 25 °C for one hour to yield drug-loaded ECnPs. The drug-loaded ECnPs were washed three times *via* centrifugation filtration (3.5 kDa MWCO) for 5 min at 15 000 rpm (21 130 rcf) each time. For the ELP-only controls of W_2_F_2_, three batches of ECnPs were also prepared *via* the same protocol, with Dex-CF : ELP mass ratios of 1 : 1, 1 : 6, and 1 : 10. The dimensions of nanostructures in each of these nine samples were obtained by measuring the average diameters of particles at temperatures from 4 °C to 80 °C, at an interval of 3 °C. Samples were incubated at each temperature for 5 minutes prior to data collection. The reported data represent an average of at least three measurements with the standard error of the mean (SEM) reported.

### Transmission electron microscopy (TEM)

Solutions for TEM were prepared for drop-casting on carbon-coated copper grids (CF300-Cu, Electron Microscopy Sciences Inc., see below). ECnPs were dissolved in water and incubated in an 80 °C oven for 2 hours. For Y_6_-G_8_ sample sets (Y_6_-G_8_-COOH and Y_6_-G_8_), samples were dissolved in DI H_2_O with a final concentration of 0.5 mg mL^−1^, followed by the annealing process from 80 °C to 25 °C as mentioned above. The pH of the sample solutions was adjusted (pH values of 1.0, 5.0, 7.0, 9.0, 10.0) using a combination of HCl and NaOH, and the pH confirmed *via* measurement on a micro pH meter (Thermo Orion Star A215). For the co-assembly studies of the Y_6_-G_8_ and Y_6_-G_8_-COOH sample sets, 250 μL of 0.5 mg mL^−1^ Y_6_-G_8_-COOH and Y_6_-G_8_ were prepared in DI water separately in two 2 mL glass vials and preheated at 80 °C for 2 hours, followed by the mixing of the two solutions at 80 °C, and cooling from 80 °C to 25 °C over 3 hours. The pH was adjusted to 5.0 using a combination of HCl and NaOH. For the Dex-CF-loaded sample sets (W_2_F_2_-G_8_), Dex-CF was predissolved in DI H_2_O at 25 mg ml^−1^ and added to the ECnP solution as described above for DLS experiments, followed by cooling to and incubation at 25 °C for 1 hour, followed by three washes with centrifugation as above.

Before TEM sample grid preparation, the grids, pipette tips, PTA (phosphotungstic acid negative stain) solution, and polypeptide solutions were incubated in an isothermal oven (VWR Signature™ Forced Air Safety Ovens, VWR Inc.) at desired temperature (25 °C, 37 °C, 50 °C, or 80 °C) for at least 1 hour. 5 μL of sample solution for TEM imaging was drop-cast on the gridat the target temperature and blotted by filter paper after 60 seconds. 3 μL of the PTA solution, also at the desired temperature, was drop-cast on the grid and blotted after 10 seconds. Three washes with DI water were employed specifically for Dex-CF/W_2_F_2_-G_8_ samples to prevent precipitation of the PTA stain. No washing steps were applied to the Y_6_-G_8_ samples to avoid altering the adjusted pH. The sample grids were kept in the oven at the desired temperature for and additional 10 min to evaporate any trace solution on the grids after filter paper blotting. TEM images were collected on a 2.1 TEM Tecnai 12 (JEOL USA Inc., Peabody, MA) at an acceleration voltage of 120 keV or on a Talos F200C at 200 keV. Each ECnP was produced and characterized three separate times to confirm the reproducibility of the results.

### Release of hydrophobic drug from ECnPs

The release profile of encapsulated Dex-CF-fluorescein from the ECnPs with different drug loading was investigated at physiological temperature. (The mass ratio of Dex-CF : ECnP was 1 : 1 (0.25 mg Dex-CF and 0.25 mg ECnP), 1 : 6 (0.042 mg Dex-CF and 0.25 mg ECnP) and 1 : 10 (0.025 mg Dex-CF and 0.25 mg ECnP).) For a 37 °C drug release experiment over 7 days, a 0.5 mL sample of Dex-CF-loaded ECnP (Dex-CF/ECnP) in solution in PBS (10 mM, pH 7.4) was dialyzed against 14 mL PBS using a 0.5 mL Slide-A-Lyzer MINI Dialysis Device (MWCO 3.5 kDa, Thermo Fisher Scientific Inc., Waltham, MA) at 37 °C. At predetermined time points, 3 mL samples were collected from the dialysis buffer sink and replenished with fresh 1× PBS. The release of Dex-CF was monitored by measuring the fluorescence intensity of the collected sample, using a PerkinElmer Fusion microplate reader (Waltham, MA, U.S.A.), with excitation/emission wavelengths of 494 nm and 518 nm. The measurements were performed at room temperature. On day 7, the Dex-CF/ECnP solution was mixed with the PBS dialysis solution and incubated at 80 °C for 30 min to fully dissociate the nanoparticles and liberate any unreleased Dex-CF for measurement. Two drug release control experiments were studied using a 1 : 1 Dex-CF/ECnP sample with 37 °C over 14 days and 50 °C over 7 days in order to assess, in more detail, the dependence of the Dex-CF/ECnP morphology on Dex-CF concentration *versus* temperature. The cumulative amount of total released Dex-CF (that was released during the experiment and added to that liberated after heating) was used as the amount of encapsulated cargo. The reported data represent an average of three individual experiments. The encapsulation efficiency (EE) (%) and loading capacity (LC) (%) were calculated as follows:EE = [(total Dex − unloaded Dex)/(total Dex)] × 100LC = [(total Dex − unloaded Dex)/(mass of ECP)] × 100

After each day of release, 5 μL of the sample solution from the dialysis device was drop-cast on a TEM grid and blotted after 60 seconds. 5 μL of the DI water solution was drop cast on the grid and blotted after 10 seconds three times to remove the salts from PBS. For staining, 1% PTA (pH adjusted to 7.0 using 1 M NaOH) as a negative stain and three washes with DI water were employed. TEM images were collected as described above in the TEM section.

## Results and discussion

### Elastin–collagen peptide selection

We have previously reported twelve W_2_F_*m*_-*b*-G_*n*_ conjugates that can self-assemble into thermoresponsive vesicle and plate-like nanostructures.^60^ Based on the assessment of the thickness of the layers observed *via* TEM in these nanostructures, the composition of the nanoparticle layers/structures is suggested to comprise the hydrophobic ELP domain in the center and hydrophilic triple-helical CLP domains at solution-exposed surfaces. In addition, we have also previously reported the potential of the ECnPs in the sequestration and controlled delivery of drugs to/from collagen-containing matrices and tissues.^21,49,51^ Here, we sought to develop a versatile ECnP system with reversible morphological transitions under different solution conditions within a single sequence and evaluate the impact of morphology on drug release.

The Y_6_-G_8_ conjugate set (Y_6_-G_8_-COOH and Y_6_-G_8_) was employed in these studies given the potential to switch morphologies owing to the inclusion of the phenol side chain and the C-terminal carboxylic acid groups, which can carry negative charges when the pH is elevated near or above its p*K*_a_. Based on previous work^61^ that showed morphological transition upon pH modification in amphiphilic polymer systems, we anticipated that the morphology of the ECnP would be possibly controlled *via* the change of the solution pH. The W_2_F_2_-G_8_ conjugates were employed in these studies given their demonstrated ability to self-assemble into thermoresponsive, plate-like morphologies in aqueous solution,^60^ and the possibility that the morphology could be sensitive to drug loading, given changes in the dimensions or ‘flexibility’ of the ELP layer. This type of behavior has been observed in previous reports^25,32,55^ showing that the feed weight ratio of a docetaxel/amphiphilic PAMAM-*b*-OEG co-dendrimer could influence the morphologies of self-assembled nanostructures. We thus anticipated that the morphology of ECnPs would be possibly controlled *via* loading of different amounts of hydrophobic drug as well. The overarching idea in our studies in total is to assess the capacity of ECCs of a single sequence to change morphology reversibly under different solution conditions, as such versatility could be useful in applications in which differential binding or trafficking of nanocarriers would be desired.

### Synthesis of ECC

**Scheme 1:**
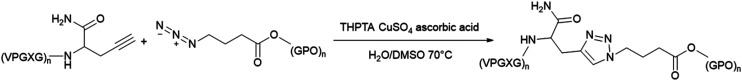
The conjugation with propargylglycine on ELP and 4-azidobutyric acid on CLP.

**Fig. 1:**
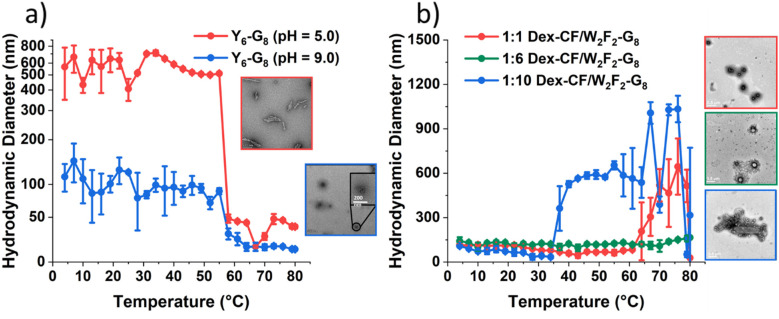
Hydrodynamic diameters (*D*_h_) of nanostructures as a function of temperature upon heating and TEM images of the self-assembled ECnPs under various solution conditions. (a) Y_6_-G_8_ in aqueous solution at pH = 5.0 (red) and pH = 9.0 (blue); (b) Dex/W_2_F_2_-G_8_ with the Dex/peptide mass ratio of 1 : 1 (red), 1 : 6 (green) and 1 : 10 (blue). The scale bars are 500 nm.

The ECCs were produced *via* copper(i)-mediated azide–alkyne cycloaddition reactions in which ELP sequences of Y_6_ and W_2_F_2_ were conjugated with CLP domains comprising G_8_ sequences. C-terminally alkyne-functionalized ELP domains and N-terminally azide-functionalized CLP domains were synthesized *via* solid-phase peptide synthesis (SPPS) methods and purified *via* reverse-phase HPLC. Then, ELPs were conjugated to the CLPs *via* copper(i)-catalyzed azide–alkyne cycloaddition as previously reported (Scheme 1).^53,56,60,62^ All bioconjugates were purified *via* HPLC, with column heating above their CLP triple-helix unfolding temperature to avoid triple-helix formation on the column. After purification *via* reverse-phase HPLC, peptides with a purity greater than 95% were obtained. ESI-MS was used to verify the purity and expected molecular mass of the peptides and ECCs (Fig. S1–S7). That Y_6_-G_8_ self-assembled into vesicle-like structures and W_2_F_2_-G_8_ self-assembled into plate-like structures at temperatures above the inverse transition temperature (*T*_t_) of the ELP domain and below the melting temperature (*T*_m_) of the CLP domain, as in our previous studies, was confirmed (Fig. S8). The *T*_t_ of Y_6_-G_8_ is below 4 °C as shown in Fig. 1, while the *T*_t_ of W_2_F_2_-G_8_ is 43 °C.^60^ The *T*_m_ of Y_6_-G_8_ is 58 °C, and the *T*_m_ of W_2_F_2_-G_8_ is 57 °C (Fig. S8). After alteration of the pH of solutions of the Y_6_-G_8_ ECnVs and loading of different amounts of Dex-CF in the W_2_F_2_-G_8_ ECnPs, the morphology, thermoresponsive properties were characterized.

### Triple helix formation by the ECC collagen domain

**Scheme 2:**
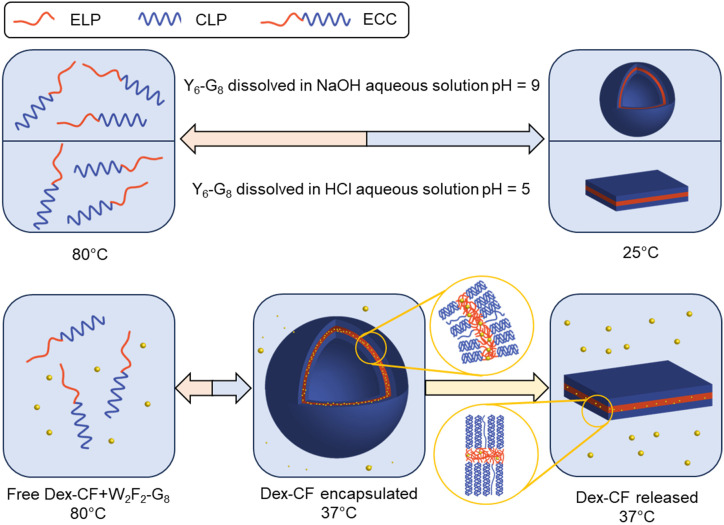
ECC self-assembly and morphologies under different solution conditions. Y_6_-G_8_ forms vesicles and plate-like structures under different pH (top) and W_2_F_2_-G_8_ shows morphological transitions dependent on the extent of drug loading (bottom).

The triple helix conformation adopted by the CLP domain in the Y_6_-G_8_ and Dex-CF/W_2_F_2_-G_8_ was confirmed *via* CD spectroscopy. One type of sample of Y_6_-G_8_ in deionized water adjusted with sodium hydroxide to pH 7 and three separate samples of Dex-CF/W_2_F_2_-G_8_ with 0.5 mg mL^−1^ total ECnP concentration were prepared. Dex-CF : ECnP solutions with mass ratios of 1 : 1, 1 : 6 and 1 : 10 were incubated at 4 °C overnight before the measurements. Full-wavelength scans, from 200 to 250 nm, were obtained for Y_6_-G_8_; the clear maximum at 224 nm at 4 °C indicated that the CLP domains in this ECC are capable of forming a triple helix. The unfolding behavior of the triple helix was monitored with the reduction of the intensity of the peak at *ca.* 224 nm upon heating (Fig. S8). Both of the Dex-CF add before the particles formation (Dex-CF/ECnP) and after the particles formation (Dex-CF + ECnP) were applied to be scanned with the 200 nm to 250 nm circular dichroism. The results from these studies with different mass ratio (1 : 1, 1 : 6, and 1 : 10) are presented in Fig. S9–S11. For ECnPs, Dex-CF/ECnP, and the Dex-CF + ECnP solutions (at a Dex-CF : peptide mass ratio 1 : 10), the clear maximum at 224 nm at 4 °C indicated that the CLP domains are capable of forming a triple helix. The unfolding behavior of the triple helix was monitored with the reduction of the intensity of the peak at *ca.* 224 nm upon heating (Fig. S9). The first derivative of the melting curve (red curve) suggested similar melting temperature (*T*_m_) for both Dex-CF-containing samples. (*T*_m(Dex-CF/W_2_F_2_-G_8_)_ = 50.8 °C, *T*_m(Dex-CF+W_2_F_2_-G_8_)_ = 53.8 °C.) Similar results were shown in the sample of Dex-CF/ECnP and Dex-CF + CLP with mass ratio of 1 : 6 (Fig. S10, *T*_m(Dex-CF/W_2_F_2_-G_8_)_ = 55.9 °C, *T*_m(Dex-CF+W_2_F_2_-G_8_)_ = 56.0 °C). The CD data for the CLP triple helix formation for the different mass ratio shows no significant changes in the mean residue ellipticity (*θ*) between 1 : 10 Dex-CF : ECnP (*θ*_224 nm_∼0.8 × 10^−3^ deg cm^2^ dmol^−1^) and 1 : 6 Dex-CF : ECnP (*θ*_224 nm_∼0.8 × 10^−3^ deg cm^2^ dmol^−1^). However, 1 : 1 Dex-CF : ECnP samples exhibit a lower signal-to-noise ratio (Fig. S11) potentially due to hydrogen binding interactions between Dex-CF and CLP (Scheme 2).^54^

### Modulating ECnP assembly with different solution conditions

DLS studies were conducted to characterize the temperature sensitivity of the assembly of different samples, including Y_6_-G_8_ at various pH (5.0, 9.0), Dex-CF-loaded W_2_F_2_-G_8_ in which Dex-CF was included in solution during self-assembly (Dex-CF/W_2_F_2_-G_8_), samples in which the Dex-CF was added to previously self-assembled W_2_F_2_-G_8_ (Dex-CF + W_2_F_2_-G_8_), and Dex-CF-only samples to assess assembly and the resulting dimensions of any resulting nanostructures under the different solution conditions. The hydrodynamic diameter (*D*_h_) of the nanostructures was plotted as a function of temperature with heating, at temperatures ranging from 4° to 80 °C. For the Y_6_-G_8_ as shown in Fig. 1a, both samples were shown to assemble into nanoparticles above 4 °C, indicating a *T*_t_ lower than 4 °C. The indicated *D*_h_ of Y_6_-G_8_ in a pH 5.0 environment (approximately 500 nm) was larger than the indicated *D*_h_ of Y_6_-G_8_ in a pH 9.0 environment (approximately 120 nm) due largely to the differences in the morphologies of Y_6_-G_8_ in these two conditions (rather than diameter differences alone, see TEM images in Fig. 1a). A notable reduction in *D*_h_ is observed in both samples at *ca.* 55 °C and 58 °C, respectively, and is ascribed to the unfolding of the CLP domain that results in the dissolution of the Y_6_-G_8_ nanostructures. The similarity in the unfolding temperature of the two samples suggests that the triple helix of G_8_ is similarly stable in pH 5.0 and pH 9.0 solutions.

**Fig. 2:**
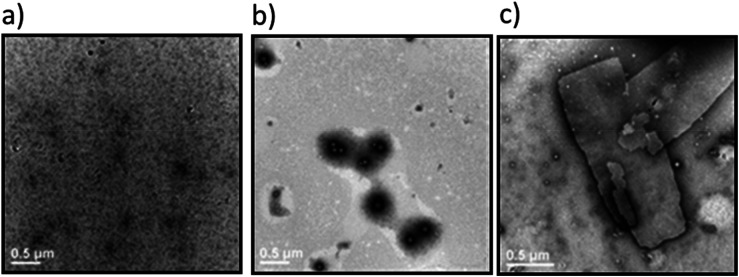
Transmission electron microscopy images of the (a) W_2_F_2_-G_8_ at 25 °C; (b) Dex-CF/W_2_F_2_-G_8_ at 25 °C; (c) W_2_F_2_-G_8_ at 50 °C. Samples were stained with 1% PTA aqueous solution. Scale bars = 500 nm.

In the case of W_2_F_2_-G_8_, *D*_h_ was monitored to evaluate the impact exerted by the presence of Dex-CF molecules. As shown in Fig. 1b, all three Dex-CF/ECnP samples (1 : 1, 1 : 6, 1 : 10 mass ratios) were shown to assemble into nanoparticles with various *D*_h_ indicated across the temperature range investigated. The diameters of 1 : 10 Dex-CF/ECnP samples (approximately 500 nm) were larger than those of 1 : 6 Dex-CF/ECnP and 1 : 1 Dex-CF/ECnP (approximately 100 nm), again likely due largely to differences in the morphologies of these samples (rather than diameter differences alone, see TEM images in Fig. 1b). For solutions in which Dex-CF was added to soluble ELP (Dex-CF + W_2_F_2_-G_8_) (Fig. S12) the DLS results indicate aggregates with *D*_h_ of approximately 200 nm, with observed values greater than 100 nm over the entire temperature range, indicating that the Dex-CF can interact with the ELP domain. This is consistent with previous reports from Rodriguez-Cabello and coworkers in which dexamethasone phosphate was shown to exhibit a perceptible effect on the ELP self-aggregation process.^57^ At high temperatures, 1 : 1 and 1 : 6 Dex-CF : ECnP samples show a marked increase in diameter, also indicating aggregation. However, the observation of light scattering in the Dex-CF-only solutions (0.25 mg Dex-CF, 0.042 mg Dex-CF and 0.025 mg Dex-CF in 0.5 mL DI H_2_O (the same Dex-CF amount as 1 : 1 Dex-CF/ECnP, 1 : 6 Dex-CF/ECnP and 1 : 10 Dex-CF/ECnP, respectively)) show aggregation of the Dex-CF over the temperature range from 4 °C to 80 °C. TEM images also showed aggregates in the Dex-CF-only solutions (Fig. S13), indicating that the inverse transition temperature of Dex-CF/ECnP cannot be easily determined *via* DLS. (TEM experiments were thus used (Fig. 2) to characterize the nanostructures at various sample preparation temperatures to roughly estimate *T*_t_.)

The lack of structures for the W_2_F_2_-G_8_ alone near room temperature (Fig. 2a) were significantly different from those observed for Dex-CF/W_2_F_2_-G_8_ ECnPs (Fig. 2b). The plate-like nanostructures observed for W_2_F_2_-G_8_ ECCs alone at 50 °C (Fig. 2c) and their absence near room temperature suggests an inverse transition temperature between these two conditions. Nanoparticles observed in Dex-CF/ECC solutions (at all ratios of Dex-CF/ECnP) were formed at temperatures below 25 °C, however, indicating that the *T*_t_ of these drug-loaded Dex-CF/ECC nanoparticles was significantly reduced relative to that observed for the W_2_F_2_-G_8_ ECnPs alone, and suggesting interaction of the Dex-CF with the ELP domain.

### Surface charge-induced morphological transformations of Y_6_-G_8_-COOH/Y_6_-G_8_ assemblies

**Fig. 3:**
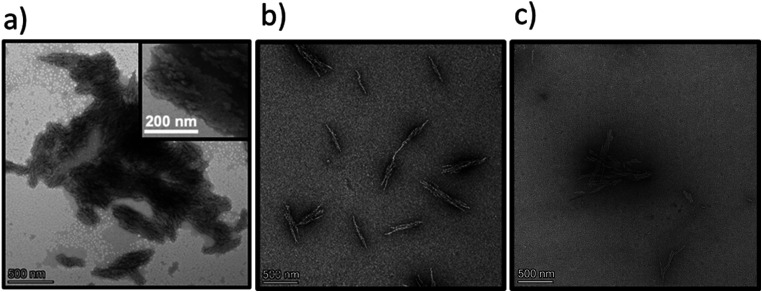
Transmission electron microscopy images for Y_6_-G_8_-COOH at various pH conditions at room temperature. (a) Y_6_-G_8_-COOH at pH 1.0; (b) Y_6_-G_8_-COOH at pH 5.0; (c) Y_6_-G_8_-COOH at pH 9.0.

As mentioned in the description of the peptide synthesis, the C-terminus of Y_6_-G_8_ comprises an amide group, rendering Y_6_-G_8_ neutral in charge at pH 7. By altering the resin used during synthesis, the C-terminus of Y_6_-G_8_ can be modified into a carboxylic acid group, which endows it with a negative charge under alkaline conditions. (To highlight this distinction, the sequence with a carboxylic acid C-terminus is referred to as Y_6_-G_8_-COOH.) Both ECCs were studied to assess possible morphological transitions induced by changes in charge in either the solvent-exposed CLP domain (G_8_-COOH) or in the collapsed ELP domain (Y_6_). The self-assembly process was initiated with an 80 °C incubation for 2 hours in DI water, followed by cooling from 80 °C to room temperature over ∼3 hours. The pH of the ECnP solution was then tuned at room temperature to the targeted pH (1.0, 5.0, 9.0), and sample grids for TEM were prepared accordingly using the method mentioned in the previous section. The representative TEM images for samples at pH 1.0, 5.0, and 9.0 are shown in Fig. 3, respectively. Plate-like structures, exhibiting dimensions of approximately 200 nm × 500 nm, were discerned at pH 1.0 (Fig. 3a). At higher pH (pH 5.0 and pH 9.0, Fig. 3b and c), ‘needle-like’ structures with a higher-aspect ratio, with dimensions of approximately 40 nm × 600 nm, were observed.

The morphological variations observed at increased pH values imply that the transition to higher-aspect ratio structures is attributable to increased surface charge, a consequence of incorporating the carboxylic acid-modified CLP sequence. Considering the p*K*_a_ of the C-terminus carboxylic acid of G to be 2.3, approximately 95% of the carboxylic acid groups of G_8_-COOH would protonated at pH 1.0 (see SI section 2). Because solvent-exposed surface of the ECnPs likely comprises the C-terminus of the CLP, the protonation of G_8_-COOH at pH 1.0 would neutralize the ECnP surface. At higher pH values (5.0, 9.0), G_8_-COOH undergoes deprotonation, resulting in increased electrostatic repulsion between CLPs that could be expected to give rise to needle-like structures with an increased curvature and surface area-to-volume ratio compared to the plate-like structures observed for the neutral ECnPs. Consistent with our observations, and with other reports, Gan *et al.* presented a chitosan-TPP (tripolyphosphate) nanoparticle system, wherein a smaller spherical morphology, exhibiting greater curvature and surface area-to-volume ratio, was observed under pH conditions that facilitated high surface charge density. Conversely, larger particles presenting a lower surface area-to-volume ratio were detected under pH conditions that yielded a more neutral charge density on the surface.^63^

**Fig. 4:**
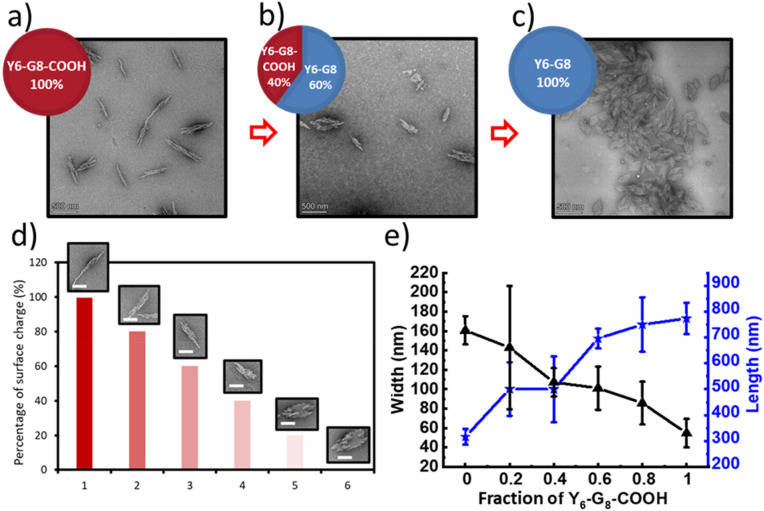
Morphological characterizations *via* transmission electron microscopy of the Y_6_-G_8_/Y_6_-G_8_-COOH co-assemblies. (a) 100% Y_6_-G_8_-COOH at RT; (b) co-assembly of 40% Y_6_-G_8_-COOH and 60% Y_6_-G_8_ at RT; (c) 100% Y_6_-G_8_ at RT; (a, b, c are all prepared in 1 mg mL^−1^ in pH 5.0 DI H_2_O, scale bars = 500 nm); (d) representative morphologies at various surface charge percentage (scale bars = 200 nm); (e) quantitative characterization of the morphologies of the ECnP co-assemblies at different Y_6_-G_8_-COOH ratios; the black curve indicates the change of the width, and the blue curve indicates the change of the length (*n* = 30).

A further experiment on co-assembly of a mixture of Y_6_-G_8_/Y_6_-G_8_-COOH was conducted at different weight ratios (100/0, 80/20, 60/40, 40/60, 20/80, 0/100) at pH 5.0; the representative TEM images are shown in Fig. 4a–c. Because Y_6_-G_8_ remains neutral at pH 5.0, and the carboxylic acid group of Y_6_-G_8_-COOH should be deprotonated, an escalation in the Y_6_-G_8_-COOH content would increase the number of negatively charged groups on the surface, thereby initiating a transition from plate-like to needle-like structures. The TEM data in Fig. 4a–c are perfectly consistent with these expectations. Fig. 4d displays observed morphologies for a given estimated surface charge, and Fig. 4e presents quantitative data related to the dimensions of the self-assembled structures at the various fractions of Y_6_-G_8_-COOH in the sample. The observed inverse relationship between surface charge and morphological aspect ratio validates the link between the surface charge and morphological transition. Several investigations have also corroborated that a high density of negative charge on the surface of cellulose nanofibrils contributes to the stabilization of these fibrils, preventing their assembly into fibril bundles or ribbons.^64–66^

### ELP hydrophilicity-induced morphological changes of Y_6_-G_8_ assemblies

**Fig. 5:**
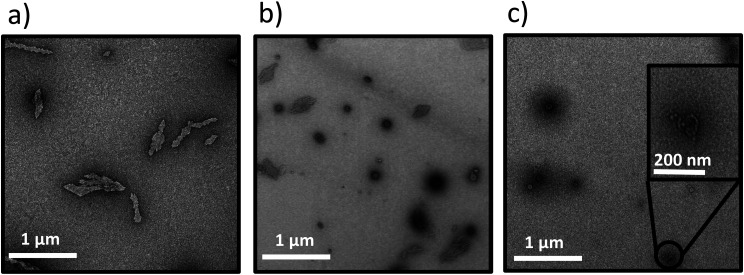
Transmission electron microscopy images for Y_6_-G_8_ ECnPs at various pH values at room temperature. (a) Y_6_-G_8_ at pH 5.0; (b) Y_6_-G_8_ at pH 7.0; (c) Y_6_-G_8_ at pH 9.0. Scale bars = 500 nm.

As noted above, the introduction of a negative surface charge onto the ECnP's results in a reversible morphological transition between high and low aspect ratios. However, the DLS measurements at various pH values shown in Fig. 1a for Y_6_-G_8_ (which lacks ionizable surface resides) also indicate a change in hydrodynamic diameter from *ca.* 500 nm (pH = 5.0) to *ca.* 120 nm (pH = 9.0). Transmission electron microscopy (TEM) measurements were thus conducted to ascertain if these changes in *D*_h_ also correlated to changes in morphology. The pH values of Y_6_-G_8_ solutions at room temperature were tuned by using a combination of 0.2 M HCl, and sample grids for TEM measurement of Y_6_-G_8_ at various pH (5.0, 7.0, 9.0) were prepared and imaged at room temperature (which is above the observed *T*_t_ for this molecule (Fig. 1a)). The representative TEM images are shown in Fig. 5a–c, where a platelet morphology (*ca.* 250 nm × 500 nm) was observed at pH 5.0, and a vesicle morphology (96 ± 10 nm) was observed at pH 9.0. In contrast, a vesicle-dominant (88.2%), mixed morphology was observed at pH 7.0 (assessed with 170 measurements of ECnPs from three TEM grids, Fig. S15) suggesting a transition from a platelet morphology to vesicle morphology with an increase in pH, which is consistent with the reduction in *D*_h_ observed in DLS measurements.

For comparison to these Y_6_-G_8_ samples, we self-assembled (VPGFG)_6_-(GPO)_8_GG (F_6_-G_8_) at pH 5.0, 7.0, and 9.0. Since the transition temperature (*T*_t_) of F_6_-G_8_ is approximately 22 °C^67^—above ambient temperature—all samples were characterized and imaged at 37 °C. F_6_-G_8_ did not exhibit any noticeable changes in particle size or morphology across the different pH conditions (Fig. S16a–c). The morphological transition observed for Y_6_-G_8_ is thus likely attributable to ionization of Y at elevated pH, although as we have reported previously,^67^ an ECnP comprising F_3_Y_3_-G_8_ did not exhibit a morphological change under similar conditions. As a control, TEM visualization of an F_2_Y_4_-G_8_ conjugate at pH 5.0 was thus conducted; a mixture of vesicle and platelet structures was observed (Fig. S17), thus corroborating the impact of Y on the morphological transitions. In comparison to F, Y has been suggested in simulations to form more extensive π–π interactions and hydrogen bonds within ECCs,^67^ which could lead to denser packing in the ELP domain. This, in turn, would reduce the ELP hydrophobic volume, and diminish the relative ELP : CLP length, supporting the adoption of a plate-like morphology.^60^ A similar observation has also been made by Helmers *et al.*, in which two amphiphilic 4,4-difluoro-4-bora-3*a*,4*a*-diaza-*s*-indacene (BODIPY) dyes with different hydrophobicity were studied, and the one with higher hydrophobicity assembled into a lamellar structure while the other one assembled into a spherical structure.^68^

The morphological change of Y_6_-G_8_ to vesicular structures at increased pH (7.0, 9.0), is thus likely a result of the ionization of the Y, which would result in the introduction of a negative charge on the Y side chain as pH values approach the p*K*_a_ (∼10.5). This would not only increase the *T*_t_ of the ELP domain,^69^ but would also expand the ELP layer, thus resulting in a vesicle structure as the ELP : CLP ratio increases^60^ at elevated pH values (inset image in Fig. 5c). A control experiment was conducted at pH 10.0, at which approximately 24% of the Y should be negatively charged (see SI), with the electrostatic repulsion and hydrophilicity causing complete dissociation of the ECnPs. Samples at pH 9.0 and pH 10.0 were reduced to pH 5.0 and incubated overnight in order to test the reversibility of this morphological change. As shown in Fig. S18, the platelet structure was recovered, consistent with reports by Dehsorkhi *et al.*, who observed a similar reversible morphological change in peptide amphiphiles (C_16_-KTTKS) from micelles (pH 2) to flat tape-like structures (pH 3).^70^ Morphological transitions of nanoparticles induced by changes in pH were also reported by Doncom *et al.*^71^ In these studies, modified *N*,*N*-diisopropylethylene diamine-functionalized polymer scaffolds bearing activated ester pentafluorophenyl acrylate (PFPA) were modified with either a charged tertiary amine acrylate or triethylene glycol methyl ether acrylate as the end group. With the introduction of highly hydrophilic groups, the polymers formed vesicles in basic aqueous solutions, transitioning into micelles under acidic conditions. This transition was reversible and reproducible. Additionally, encapsulation and release experiments with Rhodamine B, a hydrophilic dye, demonstrated that the morphological shift promoted efficient dye release under acidic conditions. In other studies, Mable *et al.* reported the development of pH-responsive ABC triblock copolymer vesicles^72^ comprising PGMA–PHPMA–PDPA triblock copolymers that were produced by extending poly(glycerol monomethacrylate) (PGMA) and poly(2-hydroxypropyl methacrylate) (PHPMA) with various lengths of the pH-sensitive polymer 2-(diisopropylamino)ethyl methacrylate (PDPA). The resulting PGMA–PHPMA–PDPA copolymer formed nanovesicles, with diameters that increased from 500 nm to 1250 nm as the pH decreased from 9 to 4 and the tertiary amine groups becamse protonated. Similarly, because of the ability of Tyr to be deprotonated (p*K*_a_ = 10.07), the morphology of the peptide Y_6_-G_8_ shifted from a plate-like structure to vesicles as the pH increased from 5 to 9. Meanwhile, the carboxylic-acid-functionalized Y_6_-G_8_-COOH transitioned from a plate-like structure to a needle-like morphology as the pH changed from 1 to 9.

### Morphological alterations triggered by drug encapsulation

To further elucidate the effects of molecular interactions on the assembled structures under different solvent conditions, W_2_F_2_-G_8_, which has been shown to adopt plate-like nanostructures,^60^ was studied. Dex-CF was encapsulated in W_2_F_2_-G_8_ ECnPs during ECnP formation as described in the materials and methods section. The release of encapsulated Dex-CF from the various Dex-CF-loaded W_2_F_2_-G_8_ was characterized by measuring the fluorescence intensity of the buffer against which the Dex-CF/ECnP samples were dialyzed. On day 7, all the samples were heated to 80 °C to dissociate the ECnPs. The drug loading efficiency was not 100% for these three samples, even after three washes. Because the DLS data were complicated by scattering of any insoluble Dex-CF, transmission electron microscopy (TEM) analysis was employed to characterize the morphologies of the Dex-CF-free ECnP samples (solely ECnP) and also Dex-CF/ECnP samples. This analysis was also employed to detect any morphological shifts of the Dex-CF/ECnP nanoparticles as a result of drug release at 37 °C (for physiological relevance) and 50 °C (a temperature exceeding the *T*_t_ of W_2_F_2_-G_8_, at which ECnPs assemble).

As reported before^60^ and shown in Fig. S8, ECnPs prepared from W_2_F_2_-G_8_ exhibited a *T*_t_ of 45 °C and *T*_m_ of 57 °C; accordingly, no assembled nanoparticles are observed at 37 °C, and plate-like nanostructures are observed at 50 °C. Interestingly, upon loading the hydrophobic drug Dex-CF, the Dex-CF/ECnP was capable of assembly at 37 °C, consistent with our DLS data and indicating the increased hydrophobicity of the Dex-CF + ELP layer, as expected. Although assembly was observed in all cases, suggesting drug interaction, the spherical morphologies of the Dex-CF-loaded W_2_F_2_-G_8_ were distinctly different from the plate-like structures observed for the unloaded W_2_F_2_-G_8_ at 50 °C. Compared with 1 : 1 Dex-CF/ECC, the 1 : 6 Dex-CF/ECC shows a similar morphology, although nanoparticles were more uniformly distributed in the solutions. In the case of the 1 : 10 Dex-CF/ECC, however, both plate-like and vesicle nanostructures were observed (*ca.* 80% vesicles and 20% plate-like nanostructures, as shown in Fig. S21a and quantified in Fig. S15b) and were more easily aggregated. These observations indicate that the degree of Dex-CF loading has a distinct impact on not only the feasibility of assembly but also offers a handle for tuning morphology.

In our previous report,^60^ the morphology of an ECnP changed from platelets to vesicles when the ratio of the length of the hydrophobic (ELP) to the hydrophilic (CLP) domain increased. We hypothesized that because of the significant high loading (LC ∼100%) of Dex-CF in the 1 : 1 and 1 : 6 Dex-CF/W_2_F_2_-G_8_ samples, the ELP bilayer increased in volume, resulting in an increased surface curvature thus promoting the formation of spherical nanostructures. For the 1 : 10 Dex-CF/W_2_F_2_-G_8_ with less Dex-CF loaded in the ELP domain, both vesicles and plate-like nanoparticles formed, potentially due to non-uniform partitioning of Dex-CF in the W_2_F_2_-G_8_. The morphologies of the Dex-CF-loaded W_2_F_2_-G_8_ at various temperatures were also investigated (Fig. S14). For 1 : 1 Dex-CF/W_2_F_2_-G_8_, assembled structures began to change morphology from smaller spherical nanoparticles (*D*_h_ ∼95 nm) to larger nanoparticles with an apparent *D*_h_ of approximately 500 nm at 50 °C; platelets appeared at 80 °C. The stability of the platelets at the elevated temperature suggests that the CLP triple helix remains intact at this temperature, which was consistent with CD data (Fig. S11) and would likely arise from the increased stability of the ELP layer afforded by drug loading. The increased stability of the ELP layer with Dex-CF loading would be minimized at lower Dex-CF concentrations, and accordingly, at 80 °C, no particle assembly was observed in either 1 : 6 Dex-CF/W_2_F_2_-G_8_ solutions or 1 : 10 Dex-CF/W_2_F_2_-G_8_ solutions. Indeed, the CD data indicated the unfolding of the CLP triple helix structure in these solutions at elevated temperatures (Fig. S9–S11).

**Fig. 6:**
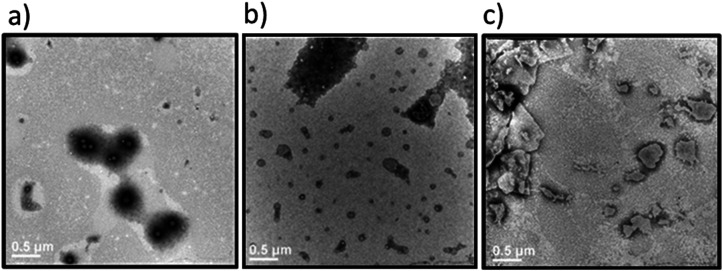
Transmission electron microscopy images of the Dex-CF/W_2_F_2_-G_8_ at various stages of drug release. Samples were stained with 1% PTA aqueous solution. (a) 1 : 1 Dex-CF/W_2_F_2_-G_8_ after washing at 37 °C; (b) 1 : 1 Dex-CF/W_2_F_2_-G_8_ after 3 days of release at 37 °C; (c) 1 : 1 Dex-CF/W_2_F_2_-G_8_ after 4 days of release at 37 °C. Scale bars = 500 nm.

**Fig. 7:**
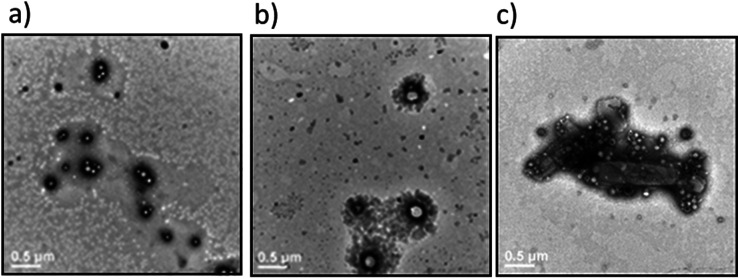
Transmission electron microscopy images of the Dex-CF/W_2_F_2_-G_8_ at 37 °C immediately after encapsulation of Dex-CF. Samples were stained with 1% PTA aqueous solution. (a) 1 : 1 Dex-CF/W_2_F_2_-G_8_; (b) 1 : 6 Dex-CF/W_2_F_2_-G_8_; (c) 1 : 10 Dex-CF/W_2_F_2_-G_8_. Scale bars = 500 nm.

The differences in morphology observed for the different Dex-CF : W_2_F_2_-G_8_ ratios suggested the possibility that a morphological transformation might also be possible over the course of Dex-CF release as well. For the 1 : 1 Dex-CF/W_2_F_2_-G_8_ sample, the ECnP retained an ECnV spherical morphology from day 0 to day 3 of drug release. The diameters of the ECnVs increased from *ca.* 95 nm (day 0) (Fig. 6a) to *ca.* 200 nm (day 3) (Fig. 6b) as the relative ratio of the lengths of the ELP and CLP domains would be decreased (with decreased loading), which is consistent with trends we reported previously.^53^ Upon full release of Dex-CF, the relative volume of the hydrophobic domain (Dex-CF + ELP), and the ELP : CLP ratio, would be further reduced, yielding larger spherical nanoparticles or plate-like nanostructures. As expected, the morphology changed from spherical to plate-like nanostructures at 4 days of Dex-CF release, after which only platelets (with dimensions of approximately 500 nm × 400 nm) were observed as additional Dex-CF was released (Fig. 6c). Across the ECnP samples loaded with different mass ratios of Dex-CF (1 : 1, 1 : 6, 1 : 10), the morphological transformations occurred on different days of release due to variations in the initial encapsulated drug amounts. The quantity of sequestered Dex-CF in the Dex-CF/W_2_F_2_-G_8_ solutions at various drug release timepoints was calculated based on the release curves. In the samples with 1 : 1 and 1 : 6 Dex-CF/W_2_G_2_-G_8_ mass ratios, ECnPs were observed to self-assembled into vesicles (Fig. 7a and b) on day 1. During the course of drug release, in 1 : 1 Dex-CF/W_2_F_2_-G_8_ solutions the morphological transition occurs when between 41 µg (day 3) and 32 µg (day 4) of Dex-CF is encapsulated in the ECnPs (Fig. S19). Similarly, in 1 : 6 Dex-CF/W_2_F_2_-G solutions, a morphological transition occurs when between 34 µg (day 1) and 29 µg (day 2) of Dex-CF is encapsulated in the ECnPs (Fig. S20). Based on the calculation, only 20 µg of Dex-CF was encapsulated in the ECnPs in the 1 : 10 Dex-CF/W_2_F_2_-G_8_ solutions. Consistent with this low Dex-CF loading, plate-like nanoparticles were observed for these ECnPs, along with nanovesicles, on day 1 (Fig. 7c) and adopted solely plate-like nanostructures on day 2 (Fig. S21). The distribution of diameters for the spherical nanoparticles is shown in Fig. S22.

Finally, to verify the solubilization of W_2_F_2_-G_8_ with the full release of Dex-CF, an extended 14-day release experiment was conducted for the 1 : 1 Dex-CF/W_2_F_2_-G_8_, and morphologies were measured *via* TEM. The dimensions of the plate-like nanoparticles formed at day 7 of Dex-CF release (500 nm × 1000 nm) decreased to 400 nm × 100 nm at day 11 (Fig. S23b). Upon complete Dex-CF release by day 14 (Fig. S23d), no structures were detectable, consistent with the behavior of the W_2_F_2_-G_8_ in the absence of Dex-CF.

**Fig. 8:**
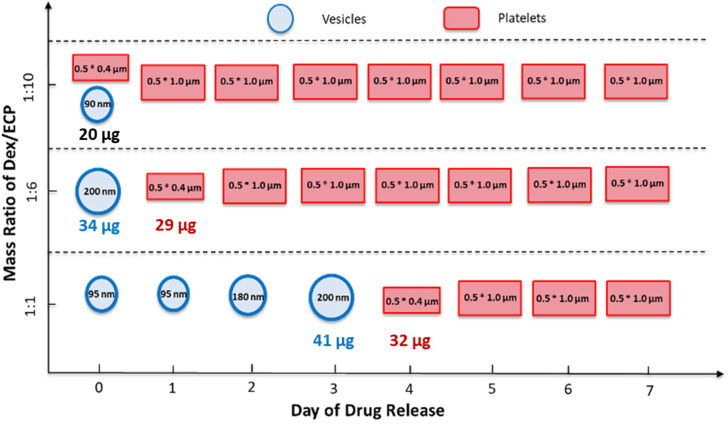
Diagram illustrating the morphological evolution of Dex-CF/W_2_F_2_-G_8_ nanoparticles at different drug-loading ratios and during drug release. For the encapsulation studies, 25 μg, 42 μg, and 250 μg of Dex-CF were added to 0.5 mL W_2_F_2_-G_8_ solutions (0.5 mg mL^−1^) to obtain 1 : 10, 1 : 6, and 1 : 1 mass ratios, respectively. After encapsulation, samples were incubated at 37 °C for over 7 days to monitor drug release. Morphologies were imaged using TEM.

The overall observations of morphological transitions for the Dex-CF-loaded W_2_F_2_-G_8_ are summarized in Fig. 8. This summary illustrates that the vesicle-forming ability of the ECCs at physiological temperature is enabled by the encapsulation of the Dex-CF. The results also highlight that the morphology of the Dex-CF-loaded nanostructures is directly dependent on the extent of the remaining Dex-CF, with a consistent and minimum level of cargo loading (*ca.* 30 mg) necessary for the formation of vesicles. Below *ca.* 30 mg of Dex-CF loading, the W_2_F_2_-G_8_ forms platelet-like nanoparticles. This morphological transition occurs at later timepoints for the 1 : 1 Dex-CF/W_2_F_2_-G_8_ owing to the greater amount of Dex-CF initially loaded in the 1 : 1 sample.

The importance of drug loading on morphology has been reported in other drug delivery systems. Studies by Guo *et al.* have shown that the feed ratio (by mass) of docetaxel to an amphiphilic PAMAM-*b*-OEG co-dendrimer can alter the morphologies of assembled nanostructures into nanosheets and nanospheres.^24^ Cao *et al.* also demonstrated that poly(1-*O*-methacryloyl-β-d-fructopyranose)-*b*-poly(methyl methacrylate) block copolymers exhibited a drug-induced morphological transition from cylindrical micelles to polymersomes when loaded with curcumin.^34^ In our study, we observed a similar transition from plate-like structures to vesicle structures upon loading of Dex-CF in ECC-based nanostructures. The increase in hydrophobic volume in the elastin-like domain due to Dex-CF loading promoted the formation of vesicles, a transition that became more pronounced as the drug concentration increased. Another comparison is with the work by Stenzel and colleagues, who showed that loading block copolymers with hydrophobic drugs not only influenced the nanocarrier morphology but also affected drug release rates.^58^ Our study similarly demonstrated that the extent of Dex-CF loading influenced both the morphology and the timescales for release of Dex-CF from ECnP nanostructures. Future studies will characterize this release in more detail for correlation with cargo uptake and efficacy.

## Conclusions

This study highlights the ability of elastin–collagen conjugates (ECCs) to undergo variable morphological transitions between vesicle and plate-like structures in response to changes in pH and also with variations in the loading of hydrophobic model drug cargo. This tunable behavior could facilitate the use of these or similar ECC molecules in the design of drug delivery systems, allowing for the adaptation of nanostructures for optimizing drug encapsulation, release profiles, and overall therapeutic efficacy. The adaptability and tunability of elastin–collagen peptide nanostructures offers myriad options for understanding of how environmental stimuli and drug loading can dictate morphology. Comparison with previous studies by other groups demonstrates that these ECC approaches may not only share the advantageous properties of polymer systems but also offer the additional benefit of biocompatibility and more precise tunability. Our studies successfully demonstrated the variable morphological transitions in ECCs under controlled *in vitro* conditions, and expanding the experimental scope to include a variety of drugs, ionic strengths, and physiological conditions will provide a broader understanding of the behavior of the ECC nanostructures. Additionally, *in vivo* studies are underway to evaluate the biocompatibility, pharmacokinetics, and therapeutic potential of drug-loaded ECCs for targeted clinical applications.

## Author contributions

H. Huang and K. Kiick conceived and designed the pH-responsive studies. J. Qin and K. Kiick conceived and designed the drug-responsive studies. H. Huang and S. Shen performed the pH-responsive experiments. J. Qin and J. Hwang performed the drug-responsive experiments. All authors analyzed the data, contributed to writing the final manuscript.

## Conflicts of interest

There are no conflicts to declare.

## Supplementary Material

BM-014-D5BM01470K-s001

## Data Availability

All the data supporting this article have been included in the main text and the supplementary information (SI). Supplementary information: ESI-MS spectra, CD spectra, DLS results and TEM imaging pictures. See DOI: https://doi.org/10.1039/d5bm01470k.
