# Supplementary material for: Fine structural tuning of the assembly of elastin–collagen peptide conjugates with drug loading and manipulation of molecular interactions
Source: Biomater Sci. 2026 May 22;14(13):3572–86. doi: 10.1039/d5bm01470k (PMC13218328; doi:10.1039/d5bm01470k)
Supplement: BM-014-D5BM01470K-s001 [file BM-014-D5BM01470K-s001.pdf]

Supplementary Information (SI) for Biomaterials Science.
This journal is © The Royal Society of Chemistry 2026

Fine structural tuning of the assembly of elastin-collagen-peptide conjugates with drug loading and tuning of molecular interactions

Haofu Huang[a,*], Jingya Qin[a,*], Sirui Shen[a,*], Jeongmin Hwang[b], Kristi L. Kiick[a,b]

[a]Department of Materials Science and Engineering, University of Delaware, Newark, DE, USA

[b]Department of Biomedical Engineering, University of Delaware, Newark, DE, USA

[*]Contributed equally

1. Solid-phase peptide synthesis

All peptides were synthesized via solid-phase peptide synthesis methods (SPPS) as described in the main content. 4-azidobutanoic acid was introduced to the N terminus of the CLP via amidation reaction. An alkyne group from propargyl glycine was attached to the C-terminus of the ELP domain via SPPS. The facile conjugation of ELP and CLP via CuAAC "click" reaction was conducted as described in the main content (Fig. 1). The molecular weight of CLP, ELPs & ELP-CLP conjugates was verified via electrospray ionization mass spectrometry (ESI-MS) After purification with reverse-phase HPLC, conjugates with purity greater than 95% were obtained.

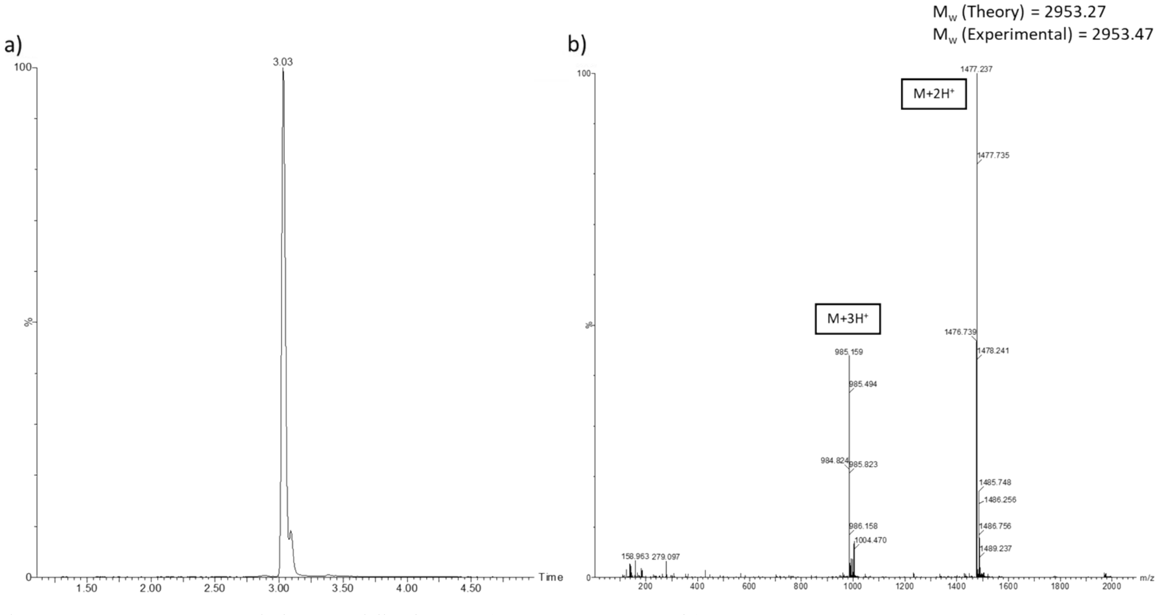

Figure S1. LC-MS of the purified Y_6: a) UPLC trace; b) ESI-MS spectrum.

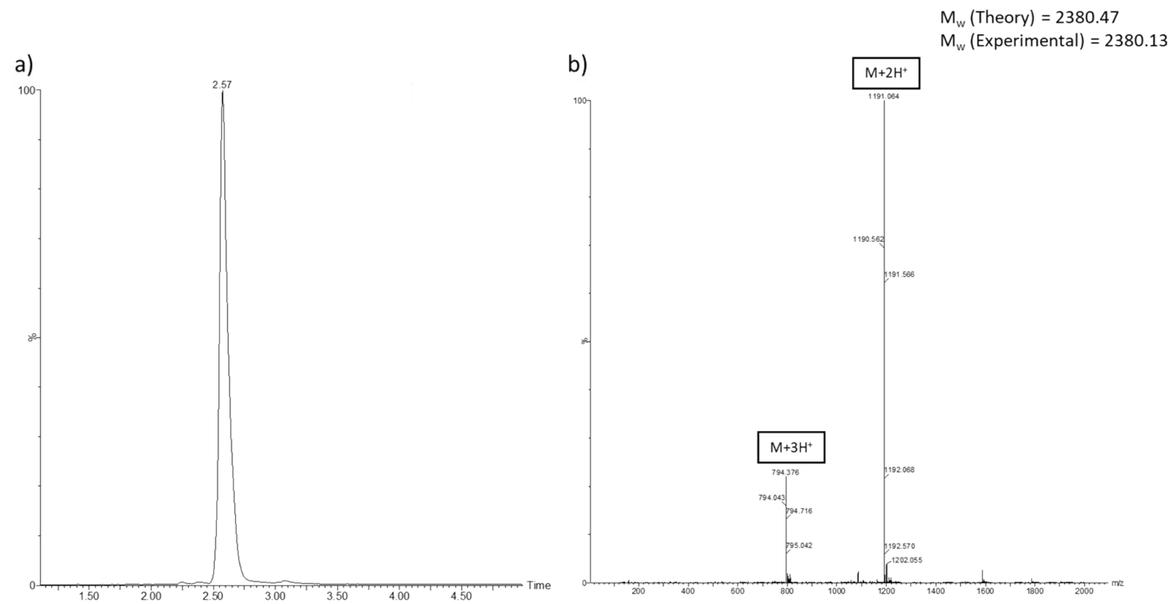

Figure S2. LC-MS of the purified G_8: a) UPLC trace; b) ESI-MS spectrum.

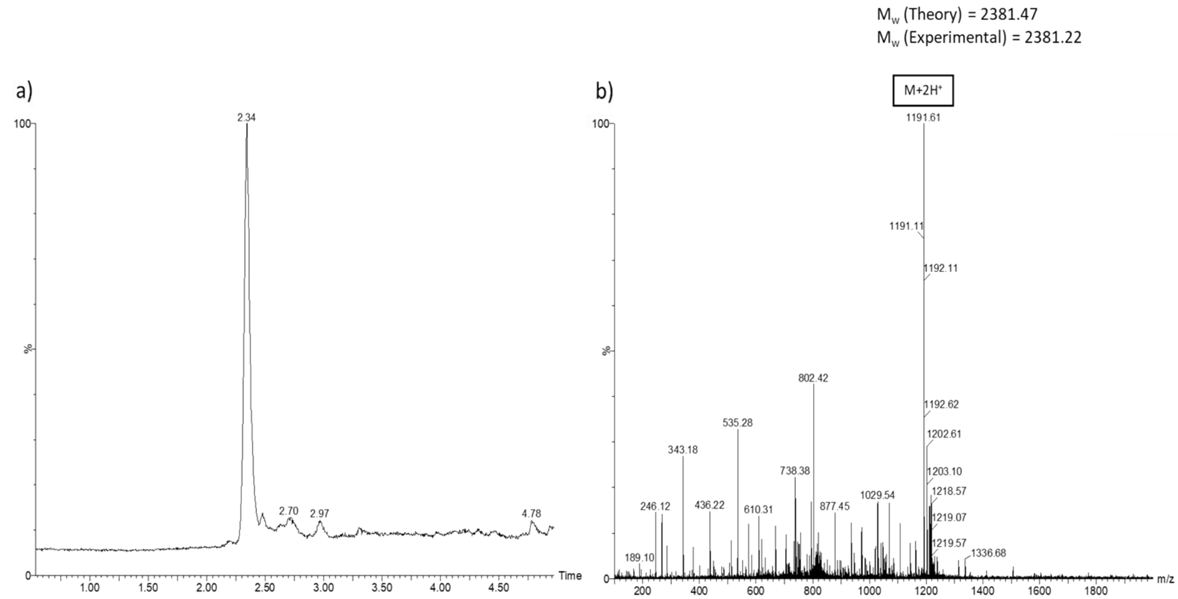

Figure S3. LC-MS of the purified G_8-COOH: a) UPLC trace; b) ESI-MS spectrum.

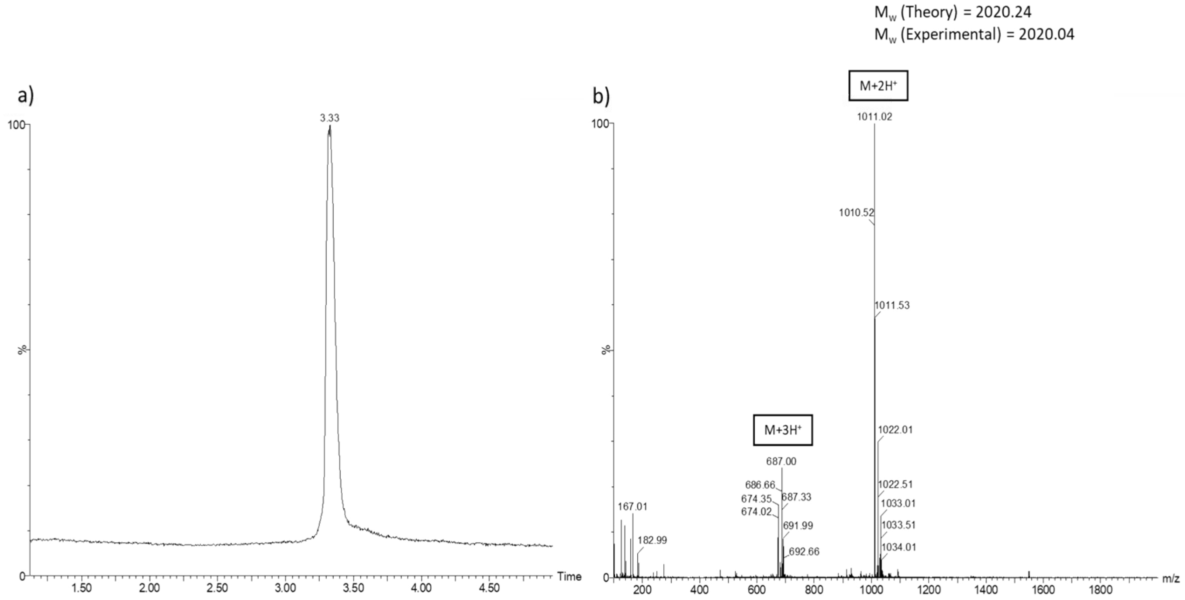

Figure S4. LC-MS of the purified W_2F_2: (a) UPLC trace; (b) ESI-MS spectrum.

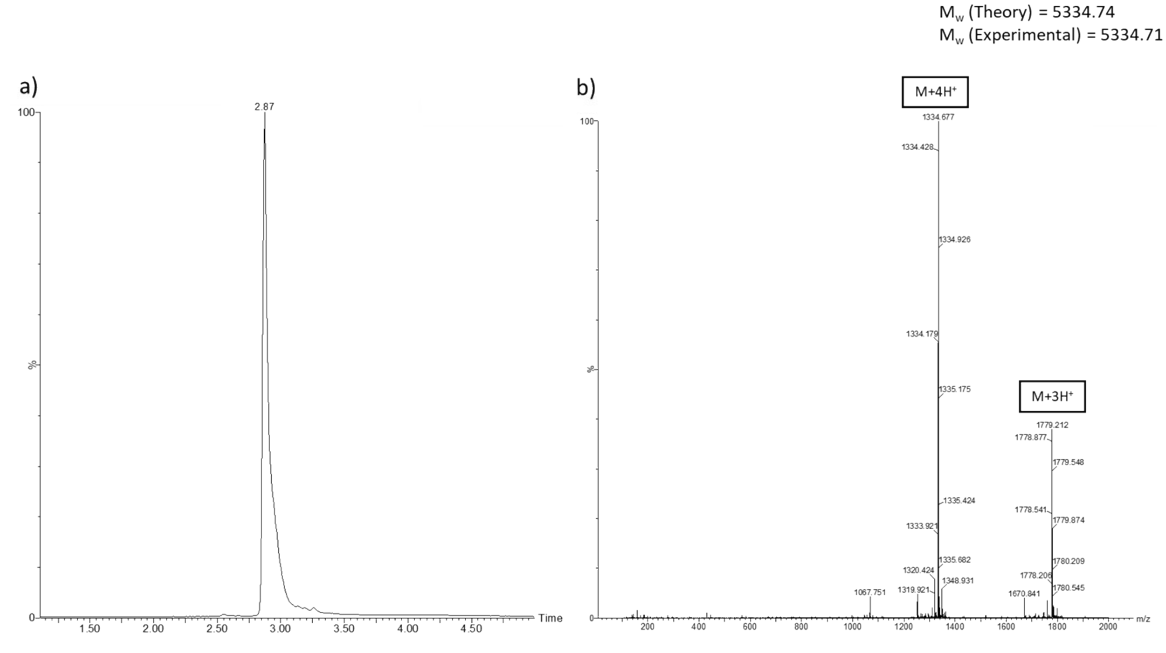

Figure S5. LC-MS of the purified Y_6-G_8-COOH: a) UPLC trace; b) ESI-MS spectrum.

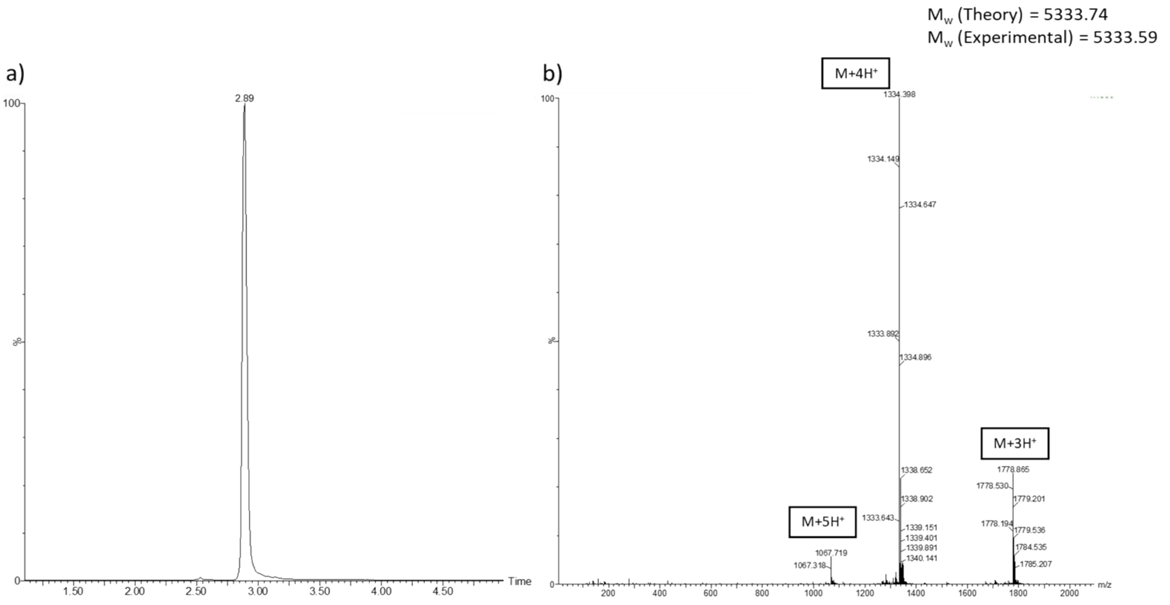

Figure S6. LC-MS of the purified Y_6-G_8: a) UPLC trace; b) ESI-MS spectrum.

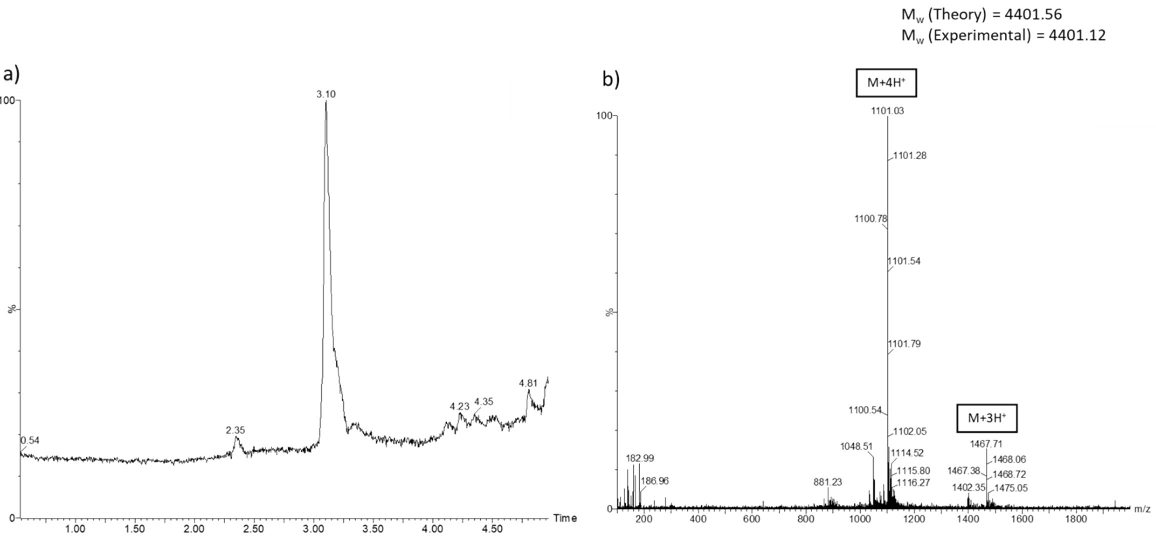

Figure S7. LC-MS of the purified W_2F_2-G_8: (a) UPLC trace; (b) ESI-MS spectrum.

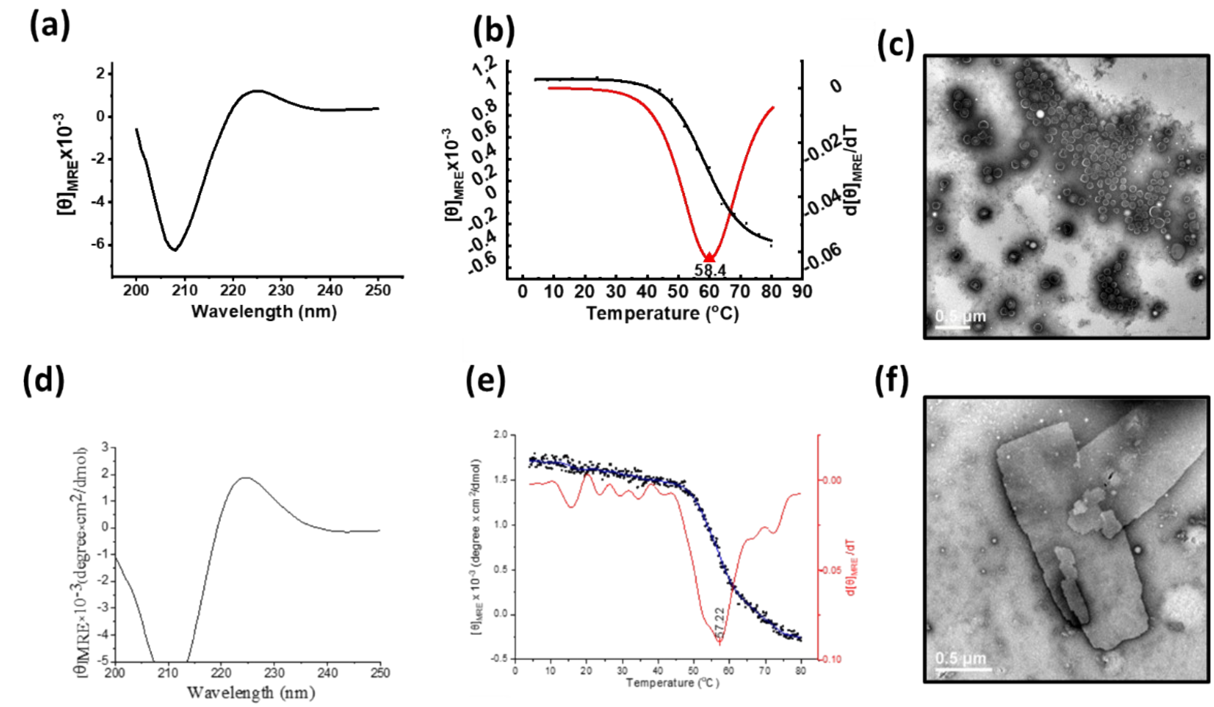

Figure S8. CD spectra and TEM measurements for W_2F_2-G_8 and Y_6-G_8. CD spectra showing representative full-wavelength scans for the (a) Y_6-G_8; (d) W_2F_2-G_8; CD spectroscopy data at 224nm for the thermal unfolding profile for the (b) Y_6-G_8; (e) W_2F_2-G_8, the first derivative of the unfolding curve with respect to temperature is shown in red; TEM images of nanostructures self-assembled from water solutions of (c) Y_6-G_8; (f) W_2F_2-G_8 at 37 °C after staining with 2% PTA.

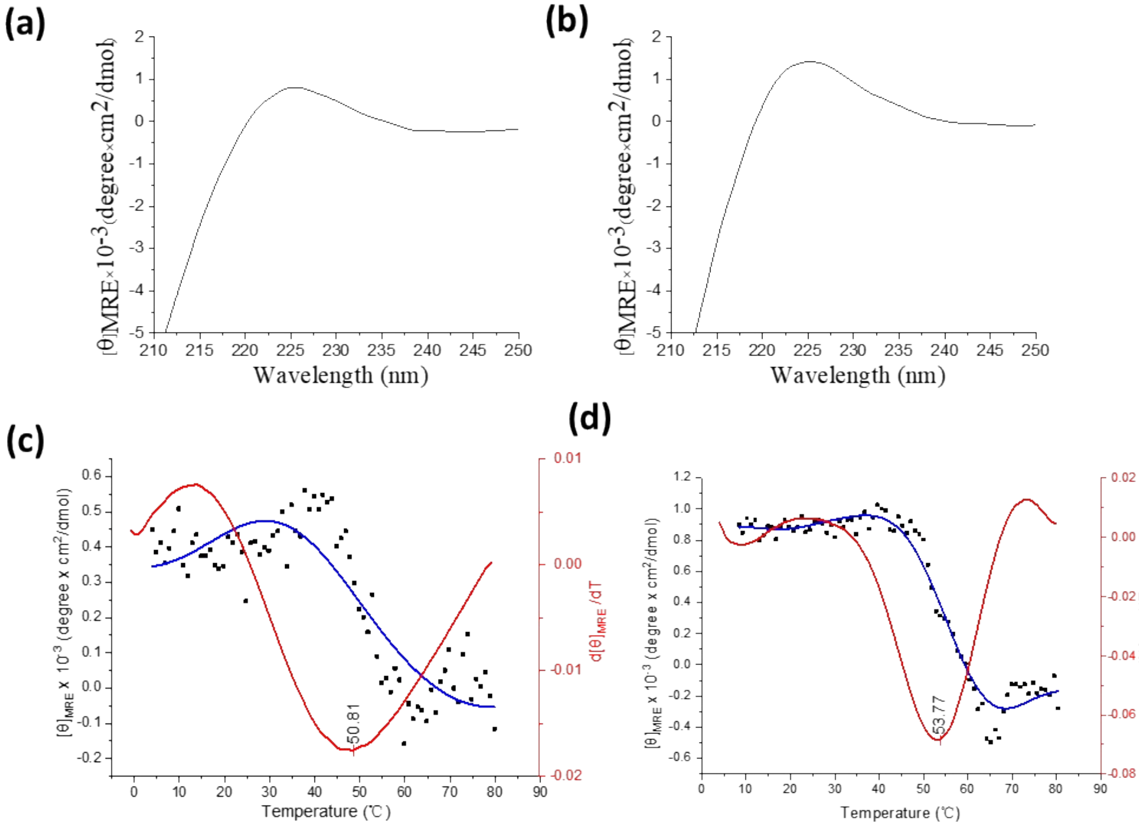

Figure S9. CD spectra showing the wavelength scans for a) Dex-CF/W_2F_2-G_8 b) Dex-CF+W_2F_2-G8 suggest folded triple helical structure. Thermal unfolding profile for c) Dex-CF/W_2F_2-G_8 d) Dex-CF+W_2F_2-G_8. Samples were mixed in solution at a 1:10 mass ratio of Dex to peptide.

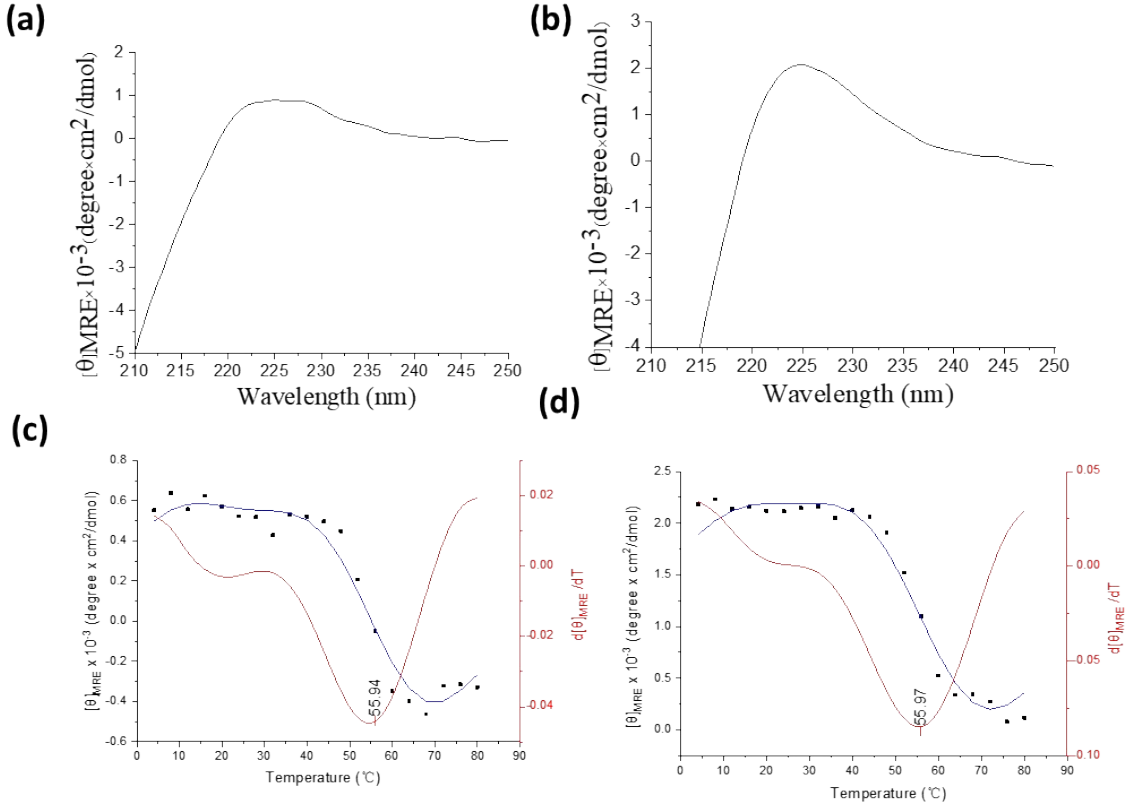

Figure S10. CD spectra showing the wavelength scans for a) Dex-CF/W_2F_2-G_8 b) Dex-CF+W_2F_2-G_8 suggest folded triple helical structure. Thermal unfolding profile for c) Dex-CF/W_2F_2-G_8 d) Dex-CF+W_2F_2-G_8. Samples were mixed in solution at a 1:6 mass ratio of Dex to peptide.

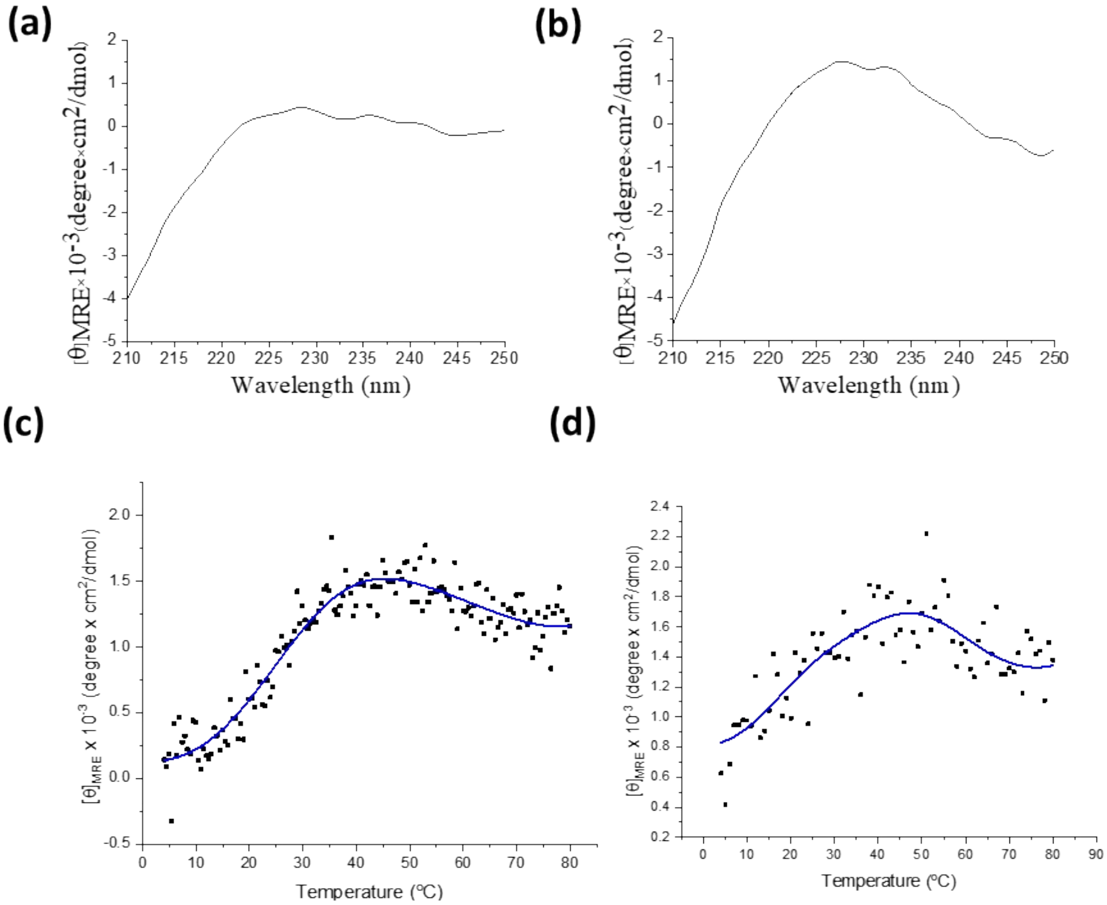

Figure S11. CD spectra showing the wavelength scans for a) Dex-CF/W_2F_2-G_8 b) Dex-CF+W_2F_2-G_8 suggest folded triple helical structure. Thermal unfolding profile for c) Dex-CF/W_2F_2-G_8 d) Dex-CF+W_2F_2-G_8. Samples were mixed in solution at a 1:1 mass ratio of Dex to peptide.

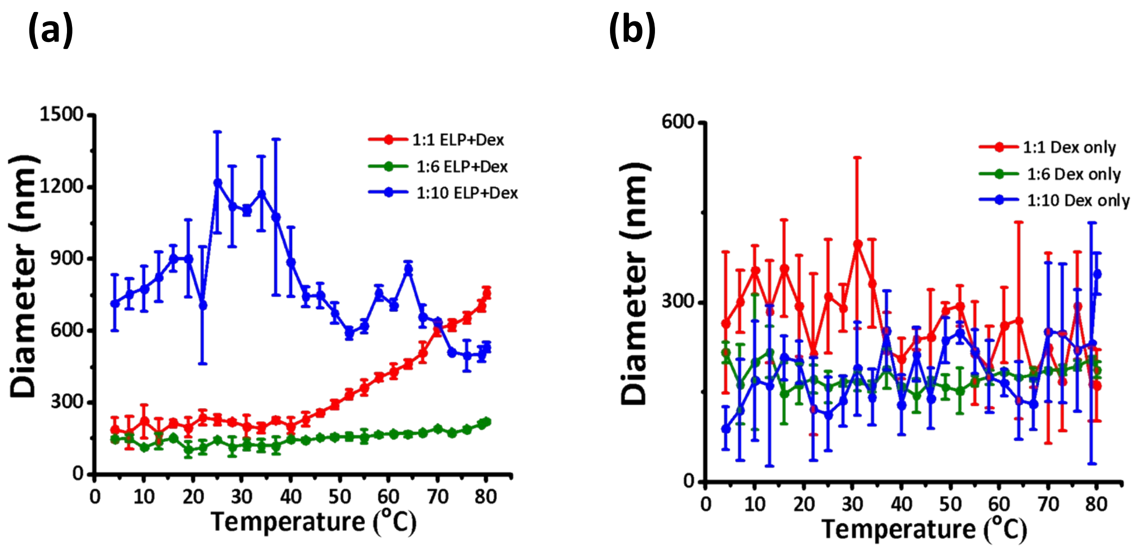

Figure S12. Dynamic light scattering results indicating the hydrodynamic diameters of nanostructures as a function of temperature upon heating. a) Dex-CF + ELP peptides mixed in mass ratio of 1:1 (0.25 mg Dex-CF), 1:6 (0.042 mg Dex-CF) and 1:10 (0.025 mg Dex-CF.; b) Drug only samples with Dex-CF concentrations equivalent to the Dex-CF:ECP samples; 1:1 (0.25 mg Dex-CF), 1:6 (0.042 mg Dex-CF) and 1:10 (0.025 mg Dex-CF).

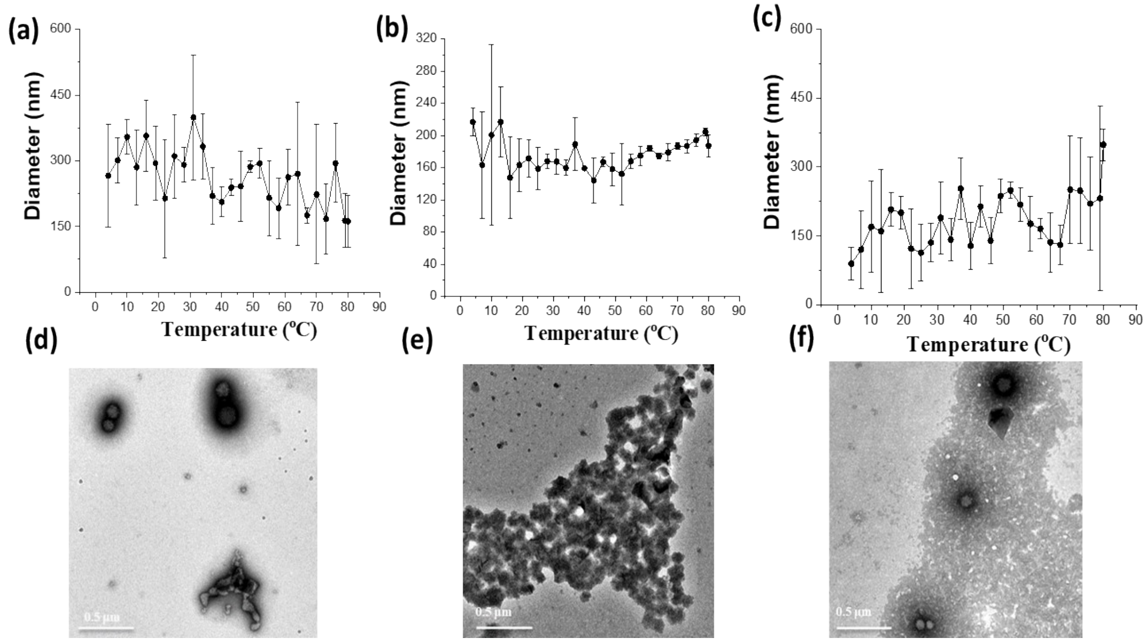

Figure S13. DLS measurements and TEM for Dex-CF-CF-only controls. a) D_h of Dex-CF only as a function of temperature upon heating (0.25 mg Dex-CF in 0.5 mL DI H_2O). b) Hydrodynamic diameters of Dex-CF only as a function of temperature upon heating (0.042 mg Dex-CF in 0.5 mL DI H_2O). c) Hydrodynamic diameters of Dex-CF only as a function of temperature upon heating (0.025 mg Dex-CF in 0.5 mL DI H_2O). d) TEM image for Dex-CF only solution (0.25 mg Dex-CF in 0.5 mL DI H_2O) after staining with 2% PTA at 37°C. e) TEM image for Dex-CF only solution (0.042 mg Dex-CF in 0.5 mL DI H_2O) after staining with 2% PTA at 37°C. f) TEM image for Dex-CF only solution (0.025 mg Dex-CF in 0.5 mL DI H_2O) after staining with 2% PTA at 37°C.

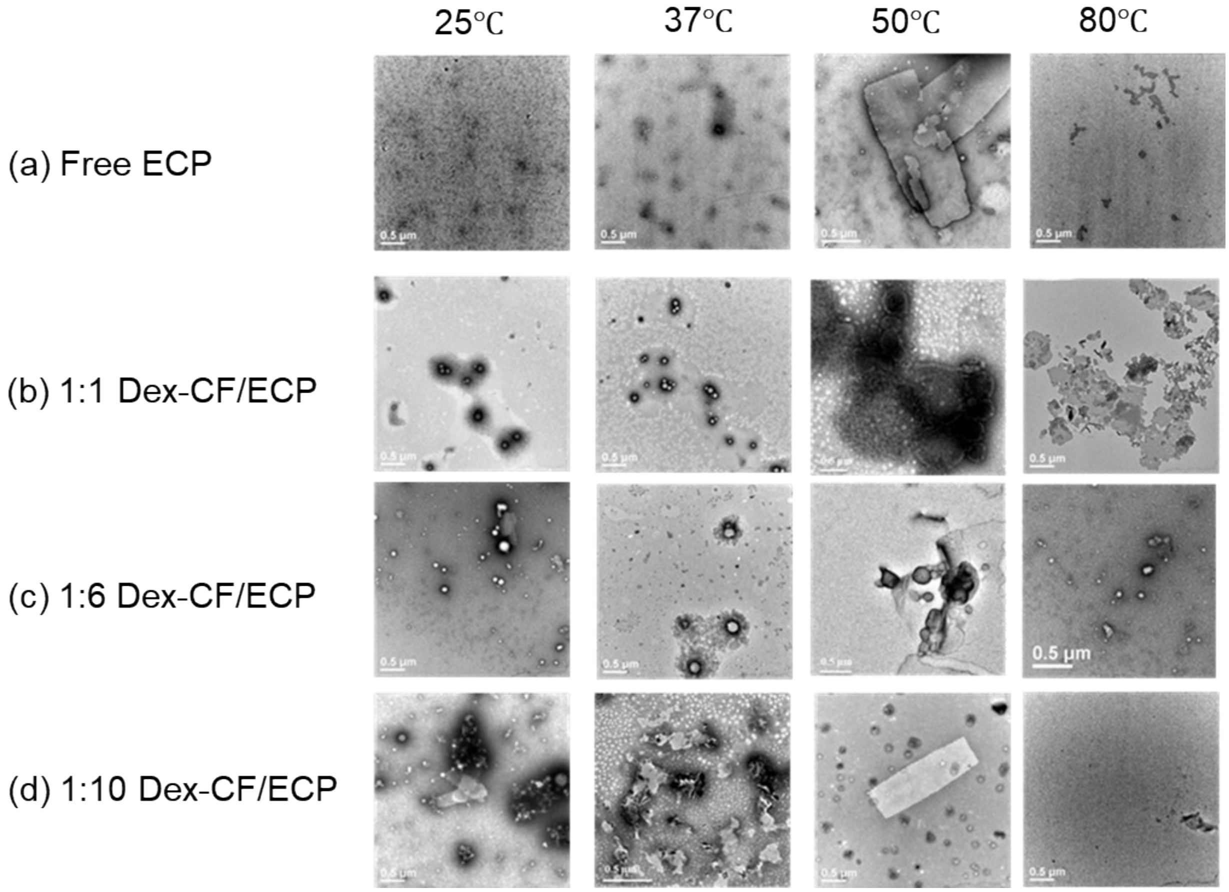

Figure S14. TEM images of nanostructures self-assembled from water solutions of Dex-CF/W_2F_2-G_8 samples at various temperatures, after staining with 2% PTA. a) W_2F_2-G_8 bioconjugates without drug loading. b) Dex-CF/W_2F_2-G_8 samples mixed in solution at a 1:1 mass ratio of Dex to peptide. c) Dex-CF/W_2F_2-G_8 samples mixed in solution at a 1:6 mass ratio of Dex to peptide. d) Dex-CF/W_2F_2-G_8 samples mixed in solution at a 1:10 mass ratio of Dex to peptide.

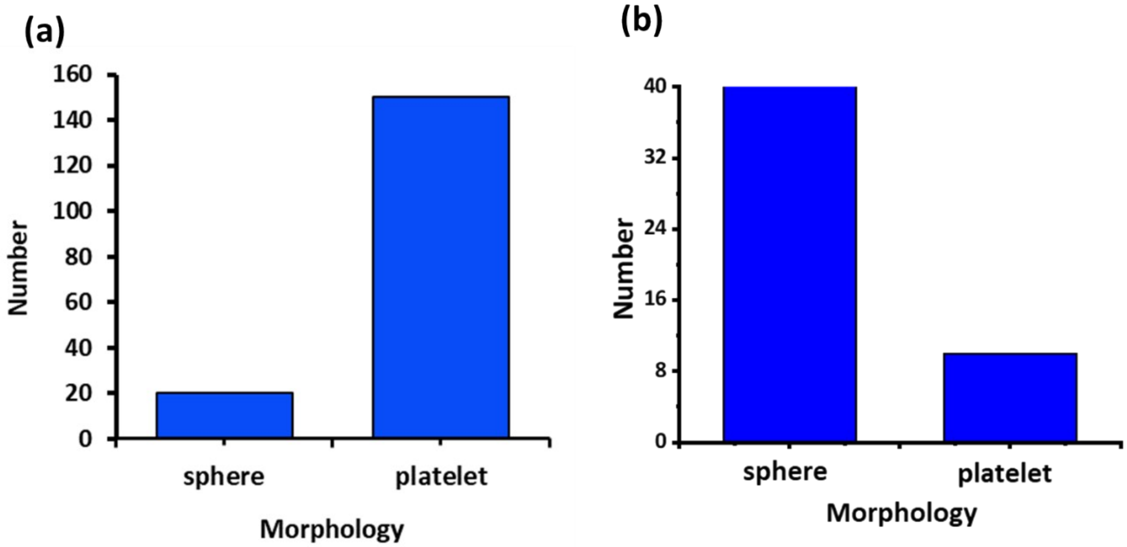

Figure S15. Quantified morphology distribution based on analysis of TEM images. (a) Y_6-G_8 at pH 7.0 with a total of 170 measurements of ECnVs taken from three grids; (b)1:10 Dex-CF/ECnV after washing at 37°C with a total of 50 measurements of ECnVs taken from one grid.

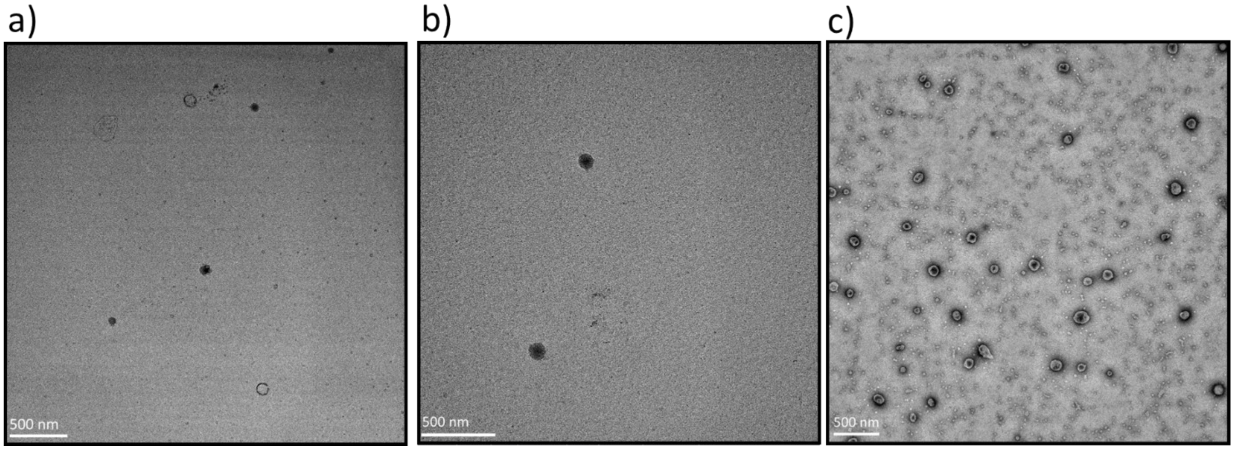

Figure S16. Transmission electron microscopy images for F-containing ECnPs with various compositions and at various pH conditions at 37 °C. a) F_6-G_8 at pH 5.0; b) F_6-G_8 at pH 7.0; c) F_6-G_8 at pH 9.0. Scale bars = 500 nm.

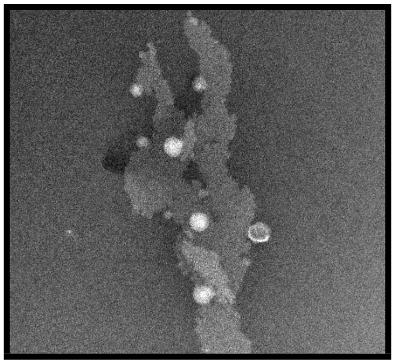

Figure S17. TEM images of F_2Y_4-G_8 at room temperature showing a mixture of morphologies of vesicles and platelets. Stained with 1% PTA,

2. The calculation of the concentration of negatively charged Y_6-G_8

Based on the K_a value of the phenol side chain of Y ($3.16e^{-11}$), the relative ratio between the negatively charged and the neutral Y is calculated based on the Henderson–Hasselbalch equation:

$$\frac{Y^-}{Y} = 10^{pH} \times 3.16 \times 10^{-11} \tag{1}$$

where ‘Y^-’ represents negatively charged Y, ‘Y’ represents none charged Y, and pH represents the pH of the solution in which the ECnV is dissolved.

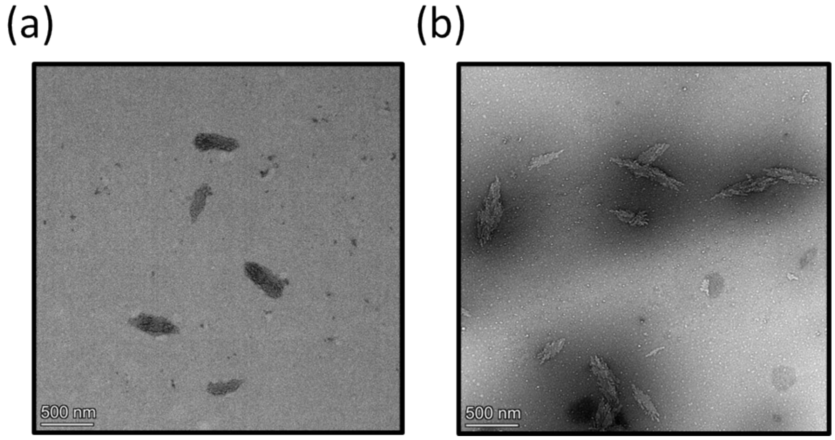

Figure S18. TEM images of Y_6-G_8 at room temperature showing the reversibility of the ECP morphologies at different pH values. a) pH=7.0; b) pH=9.0 to pH 5.0. Stained with 1% PTA, scale bars = 500 nm.

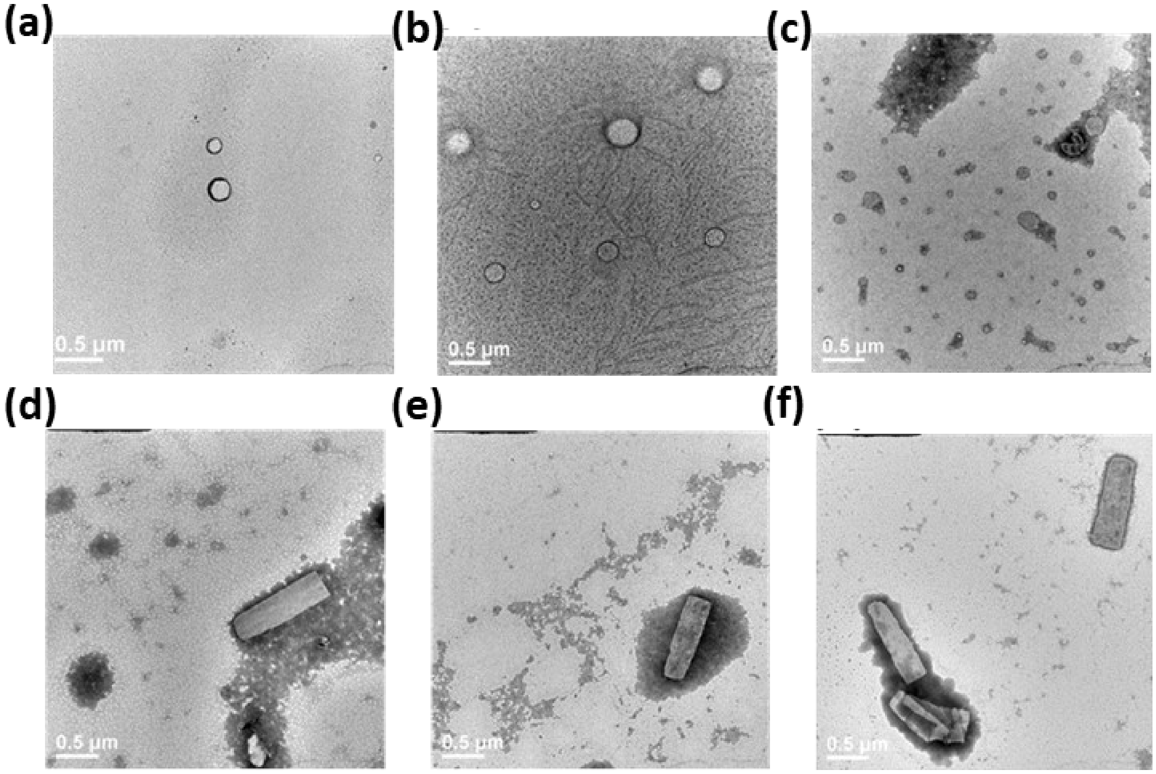

Figure S19. Transmission electron microscopy images of the 1:1 Dex-CF/W_2F_2-G_8 at various stages of drug release. 1% PTA aqueous solution (pH adjusted to 7.0) was used as a negative stain. (a) Dex-CF/ W_2F_2-G_8 samples after drug release at 1 day at 37 °C; (b) at 2 days; (c) at 3 days; (d) at 5 days; (e) at 6 days; and (f) at 7 days (all timepoints at 37 °C). Scale bars = 500 nm.

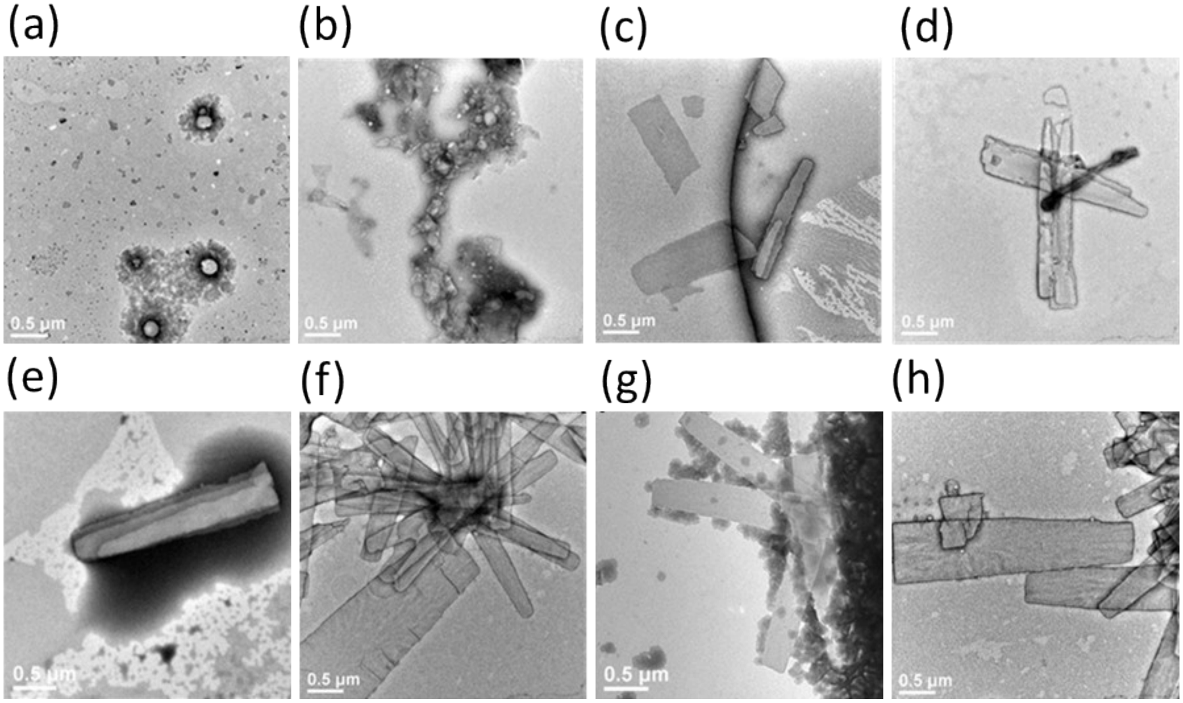

Figure S20. Transmission electron microscopy images of the 1:6 Dex-CF/W_2F_2-G_8 with drug release. 1% PTA aqueous solution (pH adjusted to 7.0) was used as a negative stain. Dex-CF/W_2F_2-G_8 samples after drug release (a) at 0 days; (b) at 1 days; (c) at 2 days; (d) at 3 days; (e) at 4 days; (f) at 5 days; (g) at 6 days; and (h) at 7 days (all timepoints at 37 °C). Scale bars = 500 nm.

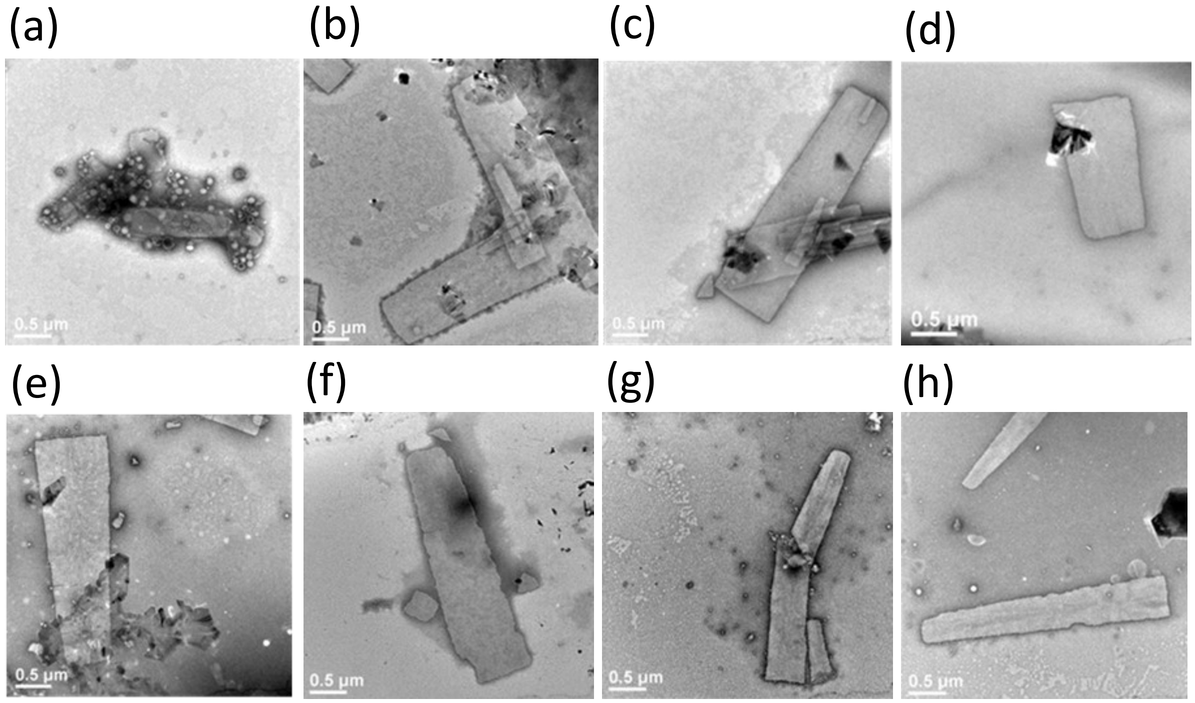

Figure S21. Transmission electron microscopy images of the 1:10 Dex-CF/W_2F_2-G_8 with drug release. 1% PTA aqueous solution (pH adjusted to 7.0) was used as a negative stain. Dex-CF/W_2F_2-G_8 samples after drug release (a) at 0 days; (b) at 1 days; (c) at 2 days; (d) at 3 days; (e) at 4 days; (f) at 5 days; (g) at 6 days; and (h) at 7 days (all timepoints at 37 °C). Scale bars = 500 nm.

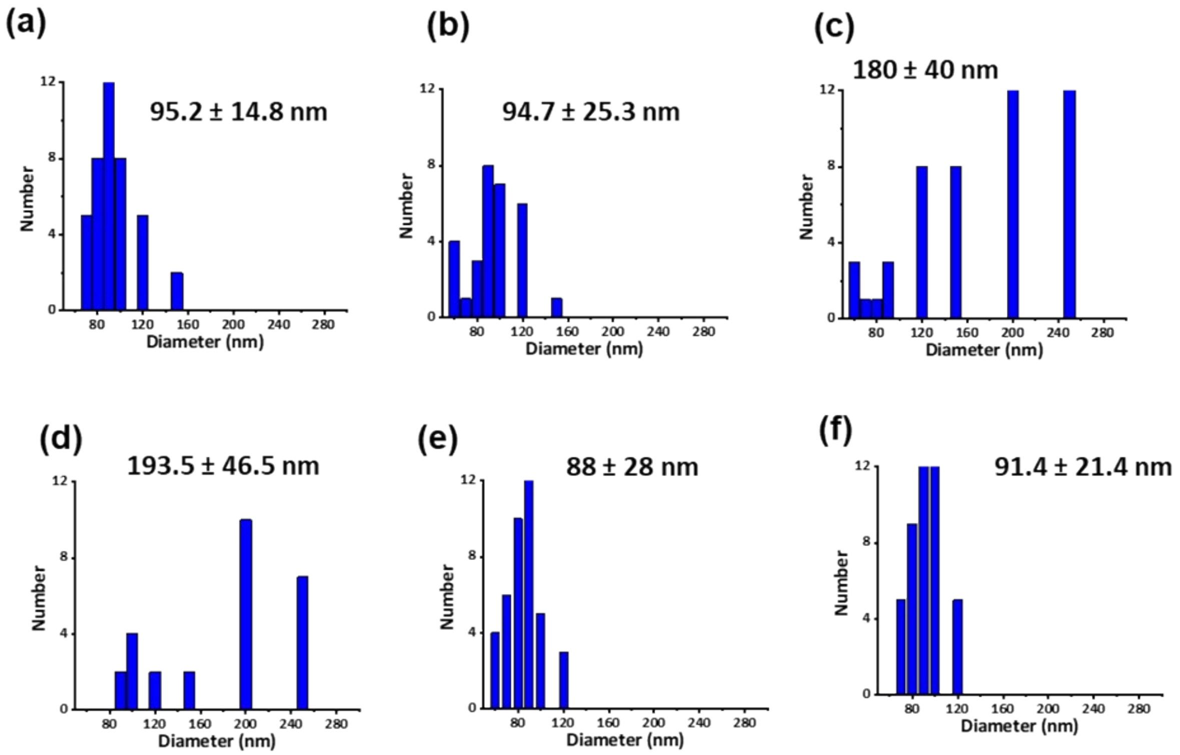

Figure S22. Vesicle diameter measurements for various Dex-CF/W_2F_2-G_8 samples at 37°C. a) 1:1 mass ratio of Dex to peptide at day 0, b) 1:1 mass ratio at day 1, c) 1:1 mass ratio at day 2, d) 1:1 mass ratio at day 3, e) 1:6 mass ratio of Dex to peptide at day 0. (f) 1:10 mass ratio of Dex to peptide at day 0. The average thickness was determined from the TEM images analyzed. The average diameters measured in the TEM images were calculated from 40 measurements of vesicles from one grid.

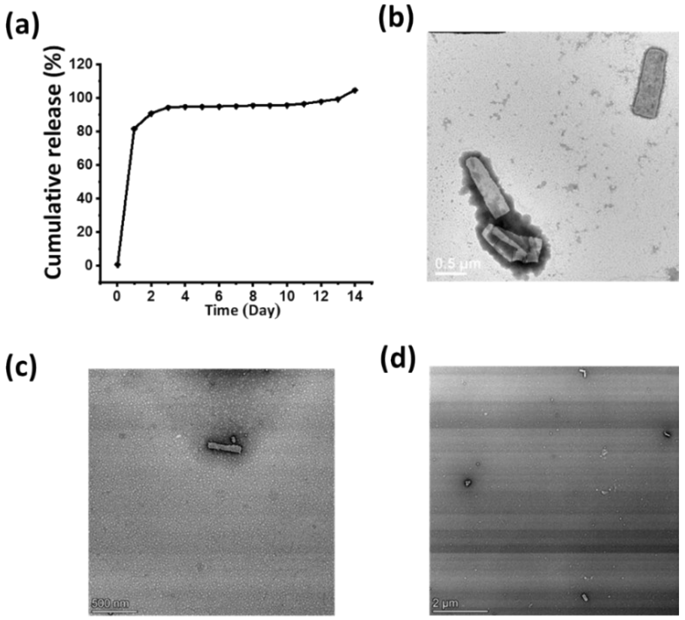

Figure S23. Morphological transition of 1:1 Dex-CF/W_2F_2-G_8 measured by TEM over the 14-day release process. Stained with 1% PTA Scale bars = 500 nm. (a) Release curve (cumulative percentage release (a) at 37 °C over 14 days; (b) Dex-CF/W_2F_2-G_8 after washing at 7 days; (c) at 11 days; (d) at 14 days.

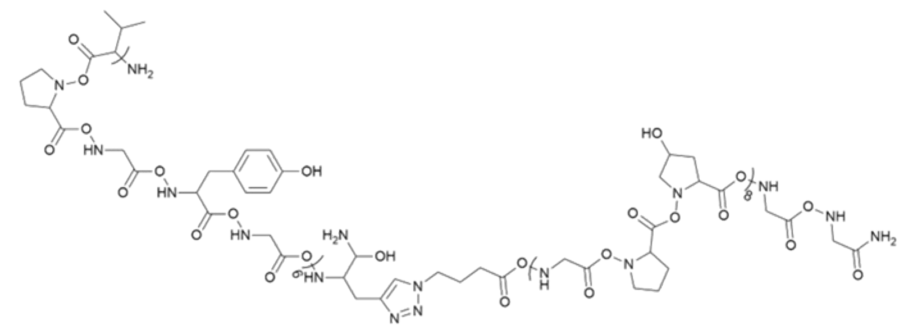

Figure S24 Y_6-G_8 chemical scheme

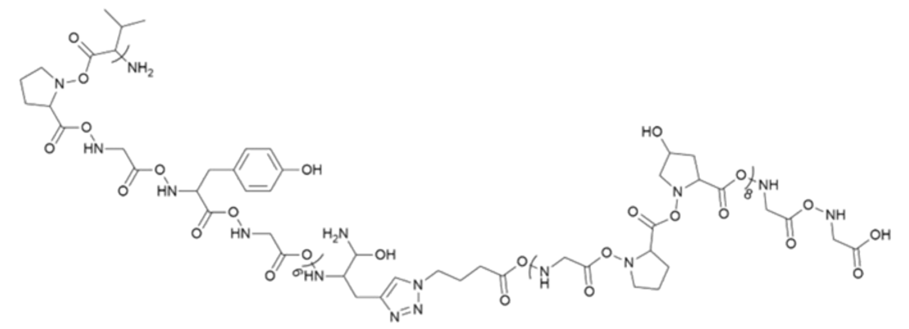

Figure S25 Y_6-G_8-COOH chemical scheme

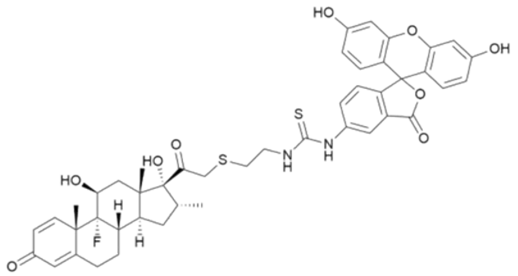

Figure S26 Dex-CF chemical scheme

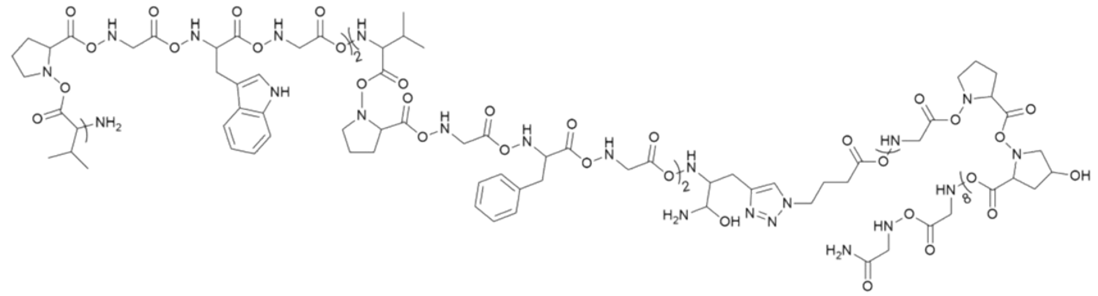

Figure S27 W_2F_2-G_8 chemical scheme
